# Remote spatial memory deficits in mouse models of neuropsychiatric disorders with immature dentate gyrus phenotype

**DOI:** 10.1093/ijnp/pyaf062

**Published:** 2025-08-23

**Authors:** Hirotaka Shoji, Hideo Hagihara, Isabella A Graef, Gerald R Crabtree, Freesia L Huang, Paul W Frankland, Tsuyoshi Miyakawa

**Affiliations:** Division of Systems Medical Science, Center for Medical Science, Fujita Health University, Toyoake, Aichi 470-1192, Japan; Division of Systems Medical Science, Center for Medical Science, Fujita Health University, Toyoake, Aichi 470-1192, Japan; Department of Pathology, Stanford University School of Medicine, Stanford, CA 94305, United States; Department of Pathology, Stanford University School of Medicine, Stanford, CA 94305, United States; Department of Genetics, Stanford University School of Medicine, Stanford, CA 94305, United States; Department of Developmental Biology, Stanford University School of Medicine, Stanford, CA 94305, United States; Howard Hughes Medical Institute, Stanford University, Stanford, CA 94305, United States; Program of Developmental Neurobiology, National Institute of Child Health and Human Development, National Institute of Health, Bethesda, MD, United States; Program in Neurosciences and Mental Health, Hospital for Sick Children, 555 University Avenue, Toronto, ON M5G 1X8, Canada; Department of Physiology, University of Toronto, Toronto, ON M5G 1X8, Canada; Department of Psychology, University of Toronto, Toronto, ON M5S 1X8, Canada; Child and Brain Development Program, Canadian Institute for Advanced Research, Toronto, ON M5G 1M1, Canada; Division of Systems Medical Science, Center for Medical Science, Fujita Health University, Toyoake, Aichi 470-1192, Japan

**Keywords:** remote memory, immature dentate gyrus, adult neurogenesis, neuropsychiatric disorders, mouse model

## Abstract

**Background:**

The hippocampal dentate gyrus (DG) is a critical region that contributes to recent and remote memory. Granule cells within this region, in which adult neurogenesis occurs, undergo dynamic and reversible maturation via genetic and environmental factors during adulthood. A pseudo-immature state of DG granule cells, called immature DG (iDG), has been observed in the adult mice of certain mutant strains, which are considered animal models of neuropsychiatric and neurodegenerative disorders, such as intellectual disability, schizophrenia, autism, and Alzheimer’s disease. However, the association between the iDG phenotype and recent and remote memories in the mouse models remains unclear.

**Methods:**

We assessed spatial memory in the Barnes circular maze task in five mutant mouse models of the disorders with the iDG phenotype, including Camk2a heterozygous knockout (HET KO), forebrain-specific Calcineurin (Cn) conditional KO (cKO), Neurogranin (Nrgn) KO, and Hivep2 (Schnurri-2) KO, and hAPP-J20 transgenic mice.

**Results:**

Camk2a HET KO mice and J20 mice spent less time around the target than their wild-type control mice in the memory retention tests 1 day and 4 weeks after the last training session. Cn cKO, Nrgn KO, and Schnurri-2 KO mice showed no significant differences in the time spent around the target from wild-type mice in the retention test 1 day after the training session, but those mutants spent less time around the target than their wild-type mice in the retest conducted 4 weeks later.

**Conclusions:**

These results indicated that mouse models of neuropsychiatric and neurodegenerative disorders exhibiting the iDG phenotype demonstrate a common behavioral characteristic of remote spatial memory deficits, suggesting the potential involvement of the pseudo-immature state of DG granule cells in remote memory dysfunction.

**Significance Statement**

A pseudo-immature state of granule cells in the hippocampal dentate gyrus (DG), called immature DG (iDG), has been observed in several mutant strains of mice considered as animal models of neuropsychiatric and neurodegenerative disorders. Thus, the iDG phenotype is proposed as one of the characteristic features of some types of the disorders. However, the impacts of iDG phenotype on hippocampal functions remains unclear. This study demonstrated that two of the five genetically modified mouse strains with iDG phenotype exhibited deficits in both recent and remote memory, with the impairments being more pronounced at the remote delay. The other three strains showed selective deficits in remote memory. These observations suggest that the pseudo-immature state of DG is associated with remote memory dysfunction, although further studies are needed to determine the causality and elucidate the underlying mechanisms.

## INTRODUCTION

The dentate gyrus (DG) is an integral part of the hippocampus that is essential for spatial learning and memory. This region is a unique structure in which new neurons are generated in the subgranular zone throughout adulthood.^1-3^ Adult neurogenesis not only supports the information and retention of new memories,^4-9^ but also drives ongoing brain plasticity. By continually integrating new neurons into existing circuits, this process can destabilize previously acquired memories, potentially contributing to their modification or forgetting.^10-18^

A growing body of evidence has shown that a “pseudo-immature state” of DG granule cells, called “immature DG (iDG)”,^19^^,^^20^ has been found in adulthood in some strains of genetically engineered mice. The “iDG” was first recognized in Camk2a heterozygous knockout (HET KO) mice,^19^^,^^20^ in which increased expressions of immature neuron markers and decreased expressions of mature neuron markers were observed in the DG, accompanying with morphological and electrophysiological features of the DG neurons similar to those of immature DG neurons in normal rodents.^21-24^ Similar molecular expression patterns in the DG were also found in human immunodeficiency virus type I enhancer binding protein 2 (HIVEP2) (also called Schnurri-2, Shn-2) KO mice,^25^^,^^26^ Neurogranin (Nrgn) KO mice,^27^ and other mutant strains of mice in adulthood.^28^^,^^29^ Furthermore, genome-wide transcriptomic analysis using microarray and RNA-seq data revealed that adult mutants– including forebrain-specific Calcineurin (Cn) conditional KO (cKO) mice,^29^ J20 transgenic mice,^30-33^ and the two other mutant strains described above–exhibit gene expression patterns in the DG that closely resemble those observed in normally developing infant wild-type mice, with respect to the number and direction of changes in immaturity-related genes. The “iDG” phenotype, defined as its similarity to overall gene expression patterns observed in the DG of developing infants, was commonly seen in the mutant strains of mice.^19^^,^^25^^,^^27^^,^^29^^,^^32^ More specifically, this phenotype is characterized by a statistically significant transcriptomic shift toward an immature neuronal state, including increased expression of genes typically enriched during early postnatal development, referred to as immaturity marker genes, and reduced expression of genes predominantly expressed in adults, referred to as mature marker genes.

Calcium/calmodulin-dependent serine/threonine protein kinase type II (CaMKII) and protein phosphatase Cn play crucial roles in neuronal signaling, synaptic plasticity, and learning and memory.^34-36^ Nrgn is a neural-specific Ca^2+^ -sensitive calmodulin-binding protein, which is involved in the regulation of CaMKII activity.^37^ Mutations and dysregulated expression of CAMK2A are associated with intellectual disability (ID), epilepsy, autism spectrum disorder (ASD), bipolar disorder, and Alzheimer’s disease (AD).^38-41^ Genetic studies have suggested associations of PPP3CA encoding the Cn α-catalytic subunit and PPP3CC encoding the Cn γ-catalytic subunit with schizophrenia^42-46^ and de novo mutations of PPP3CA in patients with ID, developmental delay, epilepsy, and autistic features.^47-52^ Large-scale genome-wide association and other studies reported that NRGN is associated with schizophrenia^53-55^ and Jacobsen syndrome with symptoms of ID.^56^ The nuclear factor-κB inhibitor HIVEP2, also known as a major histocompatibility complex-binding protein 2 (Schnurri-2, Shn-2), serves as a transcription factor that regulates several neurodevelopmental pathways.^25^^,^^57^^,^^58^ Loss-of-function variants of HIVEP2 are associated with ID and developmental delay.^59^^,^^60^ Amyloid precursor protein (APP) plays a critical role in synaptic structure and function^61^ and mutations in APP are thought to contribute to AD pathogenesis.^62^^,^^63^ J20 transgenic mice that express a mutant form of the human APP have been widely used as an AD mouse model.^30^^,^^31^ Behavioral analysis revealed that Camk2a HET KO mice,^19^^,^^64^ forebrain-specific Cn cKO mice,^29^^,^^65^ Nrgn KO mice,^66^^,^^67^ Shn-2 KO mice,^25^ and J20 mice^30^^,^^31^^,^^68^^–^^71^ exhibited shared behavioral abnormalities such as hyperlocomotor activity, abnormal social behavior, and working memory deficits, which are commonly observed in individuals with neuropsychiatric and neurodegenerative disorders, including intellectual disability, autism, schizophrenia, and/or bipolar disorder, suggesting that mutant mice with the iDG phenotype can be used as animal models of the diseases. At face value, these findings are somewhat counterintuitive given the different functions of each gene at molecular level, but regardless of different types of genetically engineered mice, all mutants had common brain phenotype, i.e., iDG, which may underlie some of common behavioral abnormalities. Molecular expression patterns similar to those of the iDG phenotype found in the mutants have been observed in the DG of patients with schizophrenia, autism, epilepsy, and AD.^72^^–^^75^ Thus, iDG has been proposed as a brain endophenotype commonly observed in certain neuropsychiatric and neurodegenerative disorders.^20^^,^^75^

The contribution of the iDG phenotype to spatial learning and memory is not well understood. Some mutant strains of mice with the iDG phenotype, such as Camk2a HET KO, Cn cKO, Shn-2 KO, and SNAP-25 knock-in mice, showed no deficits in recent reference memory that lasted for one day in spatial memory tasks using the T-maze or Morris water maze.^19^^,^^25^^,^^28^^,^^76^^,^^77^ Nrgn KO mice display impairments in water maze performance, which is suggestive of impaired recent spatial reference memory,^66^^,^^78^^,^^79^ although the nature of the deficits does not appear to be solely cognitive because maze performance might be confounded by their high anxiety-like tendencies or stress responsivity.^66^ Several studies have reported that J20 mice exhibit impaired recent reference memory in water maze tasks.^30^^,^^80^^–^^82^ Camk2a HET KO mice also showed deficits in remote memory that persisted for several weeks in the water maze task,^83^ while their remote memory in the T-maze left–right discrimination task was normal.^77^ These findings suggest that maze performance for spatial reference memory in mice with the iDG phenotype is dependent on task and retention time.

The Barnes maze test is a dry land maze test used to assess hippocampus-dependent spatial learning and memory. This is based on the natural tendency of rodents to escape from bright lights and the open space of the platform.^84^^–^^86^ This test is less stressful than the Morris water maze test,^87^ in which there is a potential danger of drowning due to water immersion and some possible confounding variables affecting maze performance, such as swimming skills and passive floating.^88^^,^^89^ Unlike the T-maze test, Barnes maze performance is not influenced by the motivational state of hunger or thirst. Thus, the Barnes maze is a suitable paradigm for providing additional opportunities evaluating the iDG phenotype in spatial memory. Genetic, pharmacological, and environmental manipulations for increasing adult neurogenesis or new immature neurons in the DG following learning promote forgetting of hippocampus-dependent memory or reduction in remote memory.^7^ Chronic treatment with fluoxetine, the selective serotonin reuptake inhibitor, inducing a “dematuration” in the DG granule cells in which mature neurons differentiate to a pseudo-immature status and re-express the molecular markers of neural progenitor cells and immature neurons^90^ as well as increased neurogenesis,^91^ caused no deficits in recent memory but impaired remote memory in the Barnes maze test.^7^ These findings led to the hypothesis that the immature state of DG granule cells is associated with the disruption of spatial memory persistence. This hypothesis remains to be tested in mutant mouse models of neuropsychiatric and neurodegenerative disorders with iDG phenotype.

In this study, we evaluated the spatial memory of five mutant mouse models of neuropsychiatric and neurodegenerative disorders showing iDG phenotype, in that, Camk2a HET KO, forebrain-specific Cn cKO, Nrgn KO, Shn-2 KO, and J20 mice (Tables 1 and 2), in the Barnes maze task to examine the possible association of iDG phenotype with recent and remote spatial reference memory. Previous studies have shown that a higher number of BrdU-positive cells in the DG was observed in Camk2a HET KO, Cn cKO, and J20 mice than in wild-type mice.^19^^,^^20^^,^^92^^–^^94^ Increased adult neurogenesis as well as the iDG phenotype may contribute to the formation and retention of spatial memory in these mutant mice. The present study assessed adult neurogenesis in the subgranular zone of the DG using BrdU labeling in Nrgn KO and Shn-2 KO mice, which has not been previously reported.

**Table 1:** Genetic mouse models of neuropsychiatric disorders showing common behavioral phenotypes

**Gene**	CAMK2A	PPP3CA/PPP3CC	NRGN	HIVEP2 (Shn-2)	APP
**Associated disease (human genetics)**	ID, ASD, BD, AD^38-41^	ID, DD, ASD^42-52^	SCZ, ID^53-56^	ID, DD^59^^,^^60^	AD^62^^,^^63^
**Mouse model**	HET KO^19^^,^^64^	KO^29^^,^^65^	KO^66^^,^^67^	KO^25^	J20^30^^,^^31^^,^^68^^–^^71^
** Locomotor activity**	↑	↑	↑	↑	↑
** Anxiety-like behavior**	↓	↓	↓	↓	↓
** Social behavior**	↓	↓	↓	↓	↓
** Motor coordination**	↑	–	↓	↑	↓
** Prepulse inhibition**	–	↓	↓	↓	↓
** Depression-like behavior**	↓	↓	↓	↓	↓
** Working memory**	↓	↓	↓	↓	↓
** Reference memory**	–	–	↓	–	↓

**Table 2:** Neurogenesis, immature dentate gyrus (iDG) phenotype, and remote memory in mutant mouse models of neuropsychiatric disorders

**Strain**		Camk2a HET KO	Cn cKO	Nrgn KO				Shn-2 KO				J20				
**Reference No**		19	20,29	27	Figure 7c	27	Figure 7d	25	25	Figure 7e,	Figure 7f,	94	92,93	30,31	32,33	92
										25	25				94	
**Age**		7–40 w	8 w–4.7 m	< 2.3 m	3.9 m	> 4.6 m	7.1 m	0.5 m	1 m	3.4–4.2 m	7.1–8.9 m	2.5 m	3 m	4–7 m	5–11 m	1 yr
**Neurogenesis marker**	BrdU	↑	↑	NE	↑	NE	→	NE	NE	↑	→	↑ (n.s.)	↑	NE	↓/→	↑
**Mature marker**	Calbindin	↓	↓	→	NE	↓	NE	→	↓	↓	↓	NE	NE	↓	NE	NE
**Immature marker**	Calretinin	↑	↑	→	NE	↑	NE	→	↑	↑	↑	NE	NE	NE	NE	NE
	DCX	↑	↑	NE	NE	NE	NE	NE	↑	↑	↑	↓	↑	NE	NE	↑
**Gene expression patterns**	Overlap similarity to developing infants^*^	*P* =	*P* = 3.7 × 10^-5^	*P* = 0.0015				*P* = 5.1 × 10^-54^				*P* = 1.9 × 10^-13^				
		4.4 × 10^-25^														
**Reference**		Figure 1	Figure 2	Figure 3				Figure 4				Figure 5				
**Age**		2.9–5.2 m	6.5–10.3 m	2.9–5.9 m				2.4–5.4 m				5.7–6.0 m				
**(probe test 1)**																
**Barnes maze test**	Learning	↓	↓	↓				→				→				
	Recent memory	↓	→	→				→				↓				
	Remote memory	↓	↓	↓				↓				↓				

w, week; m, month; yr, year; NE, not examined

^*^overlap *P*-value indicates a statistically significance in the transcriptomic shift toward an immature neuronal state, including increased expression of genes typically enriched during early postnatal development and reduced expression of genes predominantly expressed in adults.

## MATERIALS AND METHODS

The detailed methods are provided in Supplementary text.

### Animals

Five mutant strains of male mice, Camk2a heterozygous (HET) knockout (KO),^95^ forebrain-specific Cn (CNB1, Cn) homozygous KO,^96^^,^^97^ Nrgn homozygous KO, Schnurri-2 (Shn-2) homozygous KO mice,^25^^,^^58^ J20 transgenic mice,^98^ and their wild-type (WT) control mice were used. Mice were housed in plastic cages with paper bedding and given food pellets and water *ad libitum*. The room was illuminated with a 12-hour light/dark cycle (lights on at 7:00) and maintained at 23 ± 2°C. All experiments were conducted under approval of the Institutional Animal Care and Use Committee of Fujita Health University.

### Barnes Maze Task

The Barnes maze^84^ is consisted of white circular surface (100 cm diameter) with 12 holes equally spaced around the perimeter. The apparatus was placed in the sound-attenuated room and illuminated at approximately 850 lx at the center of the field. A variety of fixed extra-maze clues surrounded the apparatus. A black Plexiglas escape box was placed under one of the holes (target hole). In the training session, the mice were allowed to explore the maze freely. The training session was conducted with one to three trials per day (18 trials in total). The number of errors before first reaching the target hole, latency to reach the target hole (s), distance traveled to reach the target hole (cm), and the number of omission (defined by the visit to the target hole without subsequent entry into the target hole) were calculated. One day and approximately 4 weeks (for Nrgn KO, 28 days; for Camk2a HET KO, Shn-2 KO, and J20, 30 days; for Cn cKO, 31 days) after the last training trial, probe trials were conducted without the escape box for 180 s to assess recent memory. In the probe trials, the time spent around each hole, number of errors and latency and distance traveled to reach the target hole were calculated. To further assess spatial discrimination performance, the time spent around the target hole was compared with the time spent around adjacent holes (average time spent around the two holes positioned at both sides of the target hole) in each genotype.

### BrdU Immunohistochemistry

To assess neurogenesis in the subgranular zone of the DG in Nrgn KO mice, Shn-2 KO mice, and their WT control mice at two different ages during mature adulthood (for Nrgn, 3.9 and 7.1 months old; for Shn-2, 3.4–3.9 and 7.1–7.8 month old), mice received intraperitoneal injections of 5-bromo-2-deoxyuridine (BrdU, 50 mg/kg dose) dissolved in PBS every 2 h four times per day. Approximately 24 h after the last injection, the mice were deeply anesthetized and transcardially perfused with PBS followed by 4% PFA in 0.1 M PB. Brains were post-fixed and soaked in 30% sucrose, and were stored in a freezer until use. Brain coronal sections were boiled in 10 mM citric acid, washed with PBS, and blocked with 5% skim milk in PBS containing 0.1% Triton X-100 for 1 h at room temperature. The sections were incubated with a rat monoclonal antibody against BrdU (1:400) at 4°C overnight and incubated for 1 h with Alexa Fluor 594-conjugated anti-rat IgG (1:1000). Nuclear staining was performed with Hoechst33258. For quantitative analysis, images of at least five sections per animal were obtained using a confocal microscope and used to count the number of BrdU-positive cells in the granule cell layer of the DG. The cells were counted in a blind manner.

### Data Analysis

Behavioral data were analyzed using Student’s *t*-test, paired *t*-test, and two-way repeated-measures analysis of variance (ANOVA). The data from the BrdU assay were analyzed by Student’s *t*-test. The significance level was set at *P* < .05. Statistical analyses were performed using SAS University Edition software.

## RESULTS

The results of statistical analysis are summarized in Table 1 and Supplementary Table S1.

### Camk2a Heterozygous Knockout Mice Have Deficits in both Recent and Remote Spatial Memory

**Figure 1:**
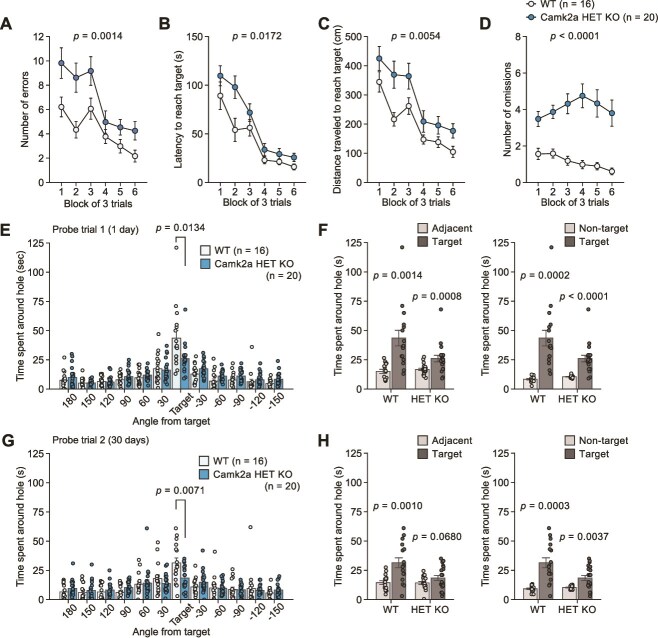
Recent and remote memory in the Barnes maze test in Camk2a heterozygous knockout mice. (**A**–**D**) Training session in Camk2a HET KO mice (n = 20) and WT mice (n = 16): (**A**) number of errors, (**B**) latency to reach the target hole (s), (**C**) distance traveled to first reach the target hole (cm), and (**D**) number of omissions. (**E**, **F**) The first probe trial conducted one day after training session: (**E**) time spent around each hole (s) and (**F**) time spent around the target hole, adjacent holes, and non-target holes (s). (**G**, **H**) The second probe trial conducted 30 days after training session: (**G**) time spent around each hole (s) and (**H**) time spent around the target hole, adjacent holes, and non-target holes (s).

In the Barnes maze test, Camk2a HET KO mice showed a significantly greater number of errors (Figure 1A), a longer latency to reach the target hole (Figure 1B), a longer distance traveled to reach the target hole (Figure 1C), and a greater number of omissions (Figure 1D) compared with WT mice. These observations indicate lower maze performance in HET KO mice during the training sessions. In the first and second probe trials, which were conducted one day and 30 days after the final training session, respectively, the time spent around the target hole was shorter in HET KO mice than in WT mice (Figure 1E), although the two genotypes of mice did not differ in each probe trial with respect to the number of errors (Figure S1A and S1D), latency to the target hole (Figure S1B and S1E), and distance traveled to reach the target hole (Figure S1C and S1F). In the probe trials, WT mice spent more time around the target hole than around the adjacent holes (Figure 1F and H) and the non-target holes (Fig. 1F and H). Although HET KO mice spent more time around the target hole than around the non-target holes in both probe trials (Figure 1F and H), there was no significant difference between the time spent around the target hole and the time spent around the adjacent holes in the second probe trial (Figure 1F and H). These data suggest that HET KO mice showed deficits for spatial discrimination at both recent and remote time points; however, the remote memory deficits were more pronounced in HET KO mice.

### Forebrain-Specific Cn Conditional Knockout Mice Have Selective Deficits in Remote Memory

**Figure 2:**
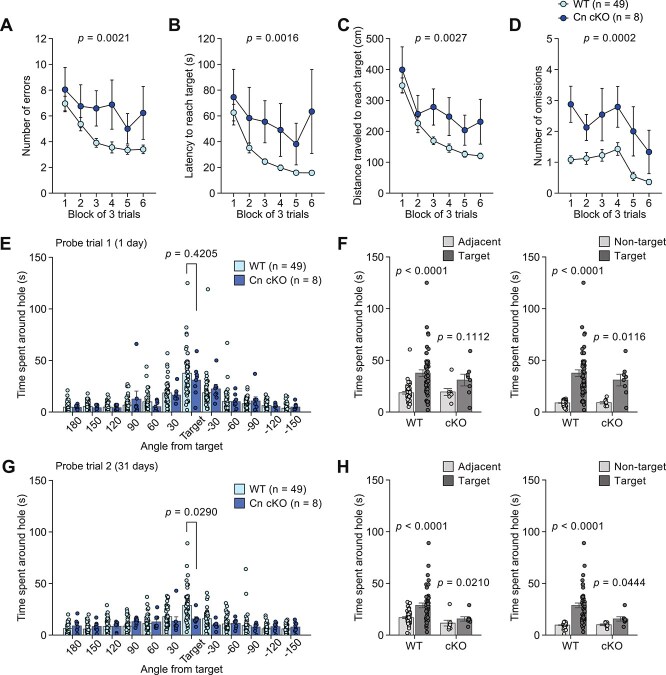
Recent and remote memory in the Barnes maze test in forebrain-specific calcineurin knockout mice. (**A**–**D**) Training session in calcineurin (Cn) cKO mice (n = 8) and WT mice (n = 49): (**A**) number of errors, (**B**) latency to reach the target hole (s), (**C**) distance traveled to first reach the target hole (cm), and (**D**) number of omissions. (**E**, **F**) The first probe trial conducted one day after training session: (**E**) time spent around each hole (s) and (**F**) time spent around the target hole, adjacent holes, and non-target holes (s). (**G**, **H**) The second probe trial conducted 31 days after training session: (**G**) time spent around each hole (s) and (**H**) time spent around the target hole, adjacent holes, and non-target holes (s).

During the training session, Cn cKO mice exhibited a greater number of errors, longer latency, longer distance traveled to reach the target hole, and a greater number of omissions than WT mice (Figure 2A–D). In the first probe trial, one day after the last training session, there was no significant difference in the time spent around the target hole between the genotypes (Figure 2E), although Cn cKO mice showed longer latency to reach the target hole than WT mice (Figure S2A–C) and exhibited reduced spatial discrimination, as shown by the lack of significant difference between the time spent around the target hole and the time spent around the adjacent holes (Figure 2F). In the second probe trial, 31 days after the last training session, mice of both genotypes spent more time around the target hole than around the adjacent holes (Figure 2H) and the non-target holes (Figure 2H), and there were no significant differences between the genotypes for the number of errors (Figure S2D), latency to the target hole (Figure S2E), and distance traveled to reach the target hole (Figure S2F). Despite the spatial discrimination, in the second probe trial, Cn cKO mice spent less time around the target hole than WT mice (Figure 2G), indicating that Cn cKO mice showed remote memory deficits.

### Neurogranin Knockout Mice Exhibit Selective Deficits in Remote Memory

**Figure 3:**
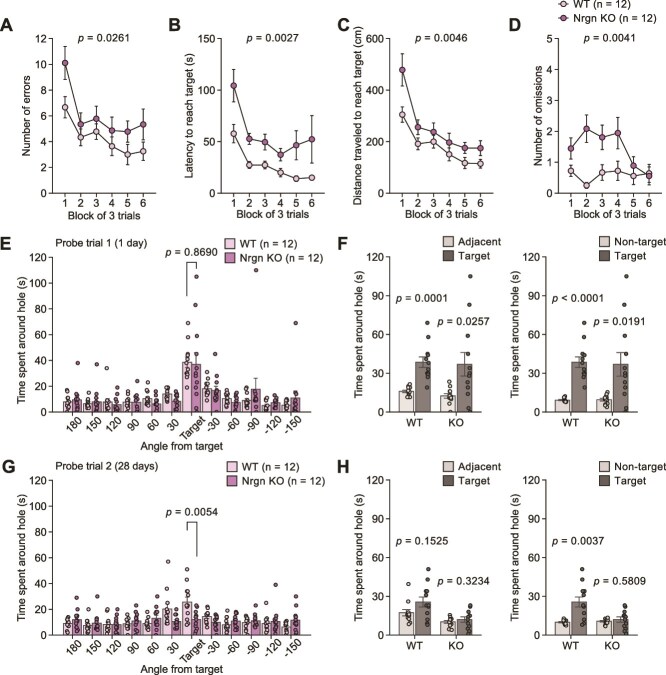
Recent and remote memory in the Barnes maze test in neurogranin knockout mice. (**A**–**D**) Training session in neurogranin (Nrgn) KO mice (n = 12) and WT mice (n = 12): (**A**) number of errors, (**B**) latency to reach the target hole (s), (**C**) distance traveled to first reach the target hole (cm), and (**D**) number of omissions. (**E**, **F**) The first probe trial conducted one day after training session: (**E**) time spent around each hole (s) and (**F**) time spent around the target hole, adjacent holes, and non-target holes (s). (**G**, **H**) the second probe trial conducted 28 days after training session: (**G**) time spent around each hole (s) and (**H**) time spent around the target hole, adjacent holes, and non-target holes (s).

Nrgn KO mice exhibited a greater number of errors, longer latency to reach the target hole, longer distance traveled to reach the target hole, and a greater number of omissions than WT mice during the training session (Figure 3A–D). In the first probe test, KO mice did not differ significantly from WT mice in the time spent around the target hole (Figure 3E), and both genotypes of mice discriminated the target hole from the adjacent holes (Figure 3F) and non-target holes (Figure 3F). There were no significant genotype differences in the number of errors, latency, or distance traveled to reach the target hole in the first probe trial (Figure S3A–C). In the second probe trial, KO mice spent less time around the target hole than WT mice (Figure 3G), and KO mice exhibited a higher number of errors, longer latency, and longer distance traveled to reach the target hole than WT mice (Figure S3D–F). KO mice also displayed no significant differences between the time spent around the target hole and the time spent around the adjacent holes (Figure 3H), and between the time spent around the target hole and the time spent around the non-target holes (Figure 3H), suggesting decreased remote memory and spatial discrimination.

### Schnurri-2 Knockout Mice Show Severe Deficits in Memory at Remote Time Point

**Figure 4:**
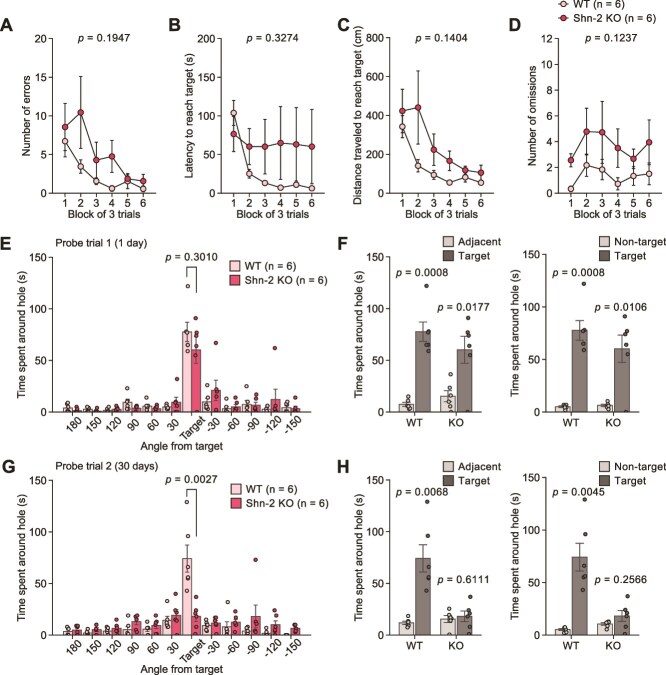
Recent and remote memory in the Barnes maze test in Schnurri-2 knockout mice. (**A**–**D**) Training session in Schnurri-2 (Shn-2) KO mice (n = 6) and WT mice (n = 6): (**A**) number of errors, (**B**) latency to reach the target hole (s), (**C**) distance traveled to first reach the target hole (cm), and (**D**) number of omissions. (**E**, **F**) The first probe trial conducted one day after training session: (**E**) time spent around each hole (s) and (**F**) time spent around the target hole, adjacent holes, and non-target holes (s). (**G**, **H**) The second probe trial conducted 30 days after training session: (**G**) time spent around each hole (s) and (**H**) time spent around the target hole, adjacent holes, and non-target holes (s).

Shn-2 KO and WT mice showed no significant differences in the number of errors (Figure 4A), distance traveled to reach the target hole (Figure 4C), or number of omissions (Figure 4D) during the training session. The latency to reach the target hole was significantly decreased across the blocks in WT mice but not in KO mice (Figure 4B). In the first probe trial, the time spent around the target hole in KO and WT mice did not differ significantly (Figure 4E), and the two genotypes of mice discriminated the target hole from the adjacent holes and the non-target holes (Figure 4F). Thirty days after the last training session, KO mice spent significantly less time around the target hole than WT mice did (Figure 4G). WT mice spent more time around the target hole than the adjacent holes and non-target holes (Figure 4H), whereas no significant differences were found between the time spent around the target hole and the time spent around the other holes in KO mice (Figure 4H). In the first and second probe trials, there were no significant genotype differences in the number of errors, latency, or distance traveled to reach the target hole (Figure S4A–F).

### hAPP-J20 Mice Exhibit both Recent and Remote Memory Deficits

**Figure 5:**
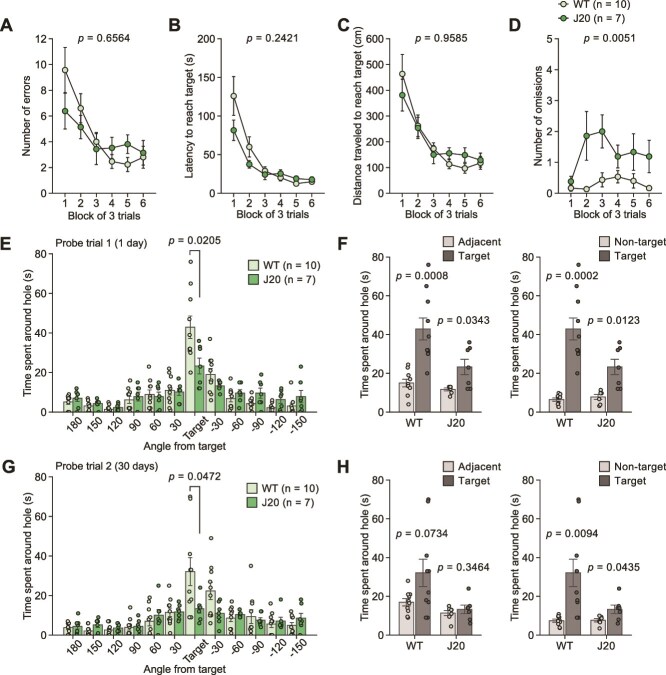
Recent and remote memory in the Barnes maze test in J20 mice. (**A**–**D**) training session in J20 mice (n = 7) and WT mice (n = 10): (**A**) number of errors, (**B**) latency to reach the target hole (s), (**C**) distance traveled to first reach the target hole (cm), and (**D**) number of omissions. (**E**, **F**) The first probe trial conducted one day after training session: (**E**) time spent around each hole (s) and (**F**) time spent around the target hole, adjacent holes, and non-target holes (s). (**G**, **H**) The second probe trial conducted 30 days after training session: (**G**) time spent around each hole (s) and (**H**) time spent around the target hole, adjacent holes, and non-target holes (s).

During the training session, J20 mice did not differ from WT mice in terms of the number of errors, latency, or distance traveled to reach the target hole (Figure 5A–C). J20 mice showed a greater number of omissions than WT mice (Figure 5D). In the first probe trial, J20 mice spent less time around the target hole than did WT mice (Figure 5E). Both genotypes of mice showed spatial discrimination in the first probe trial, as indicated by the longer time spent around the target hole than the adjacent holes and non-target holes (Figure 5F). In the second probe trial, 30 days after the training session, J20 mice spent less time around the target hole than WT mice did (Figure 5G). No significant difference in time spent around the target hole and time spent around adjacent holes was found in J20 mice, whereas WT mice showed a tendency toward longer time around the target hole than around adjacent holes (Figure 5H). A lower performance in reaching the target hole was also observed in J20 mice in the second probe trial, as indicated by the increased number of errors (Figure S5A and S5D), increased latency to the target hole (Figure S5B and S5E), and distance traveled to the target hole (Figure S5C and 5F).

### Mutant Mice Showing Good Learning Performance Exhibit Remote Memory Deficit

**Figure 6:**
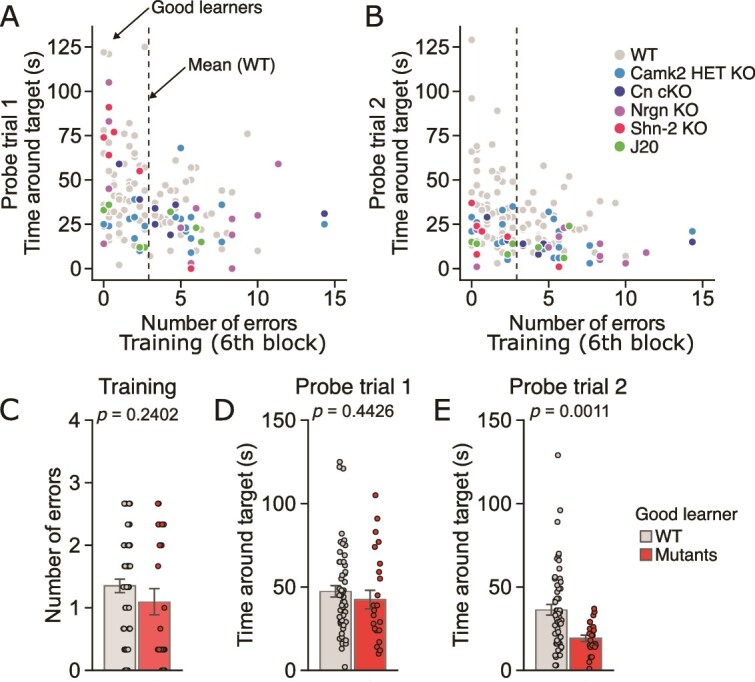
Remote memory deficit in good learners of mutant mice with immature dentate gyrus phenotype. (**A**–**B**) The scatter plots of the number of errors in the last training session and time spent around the target hole at the probe trials 1 (**A**) and probe trial 2 (**B**). The dotted lines indicate the mean value of the number of errors in wild-type control mice (mean = 2.92) of the five mutant strains. Mice showing the number of errors lower than the mean value were assigned as a good learner. (**C**) The number of errors, (**D**) time spent around the target hole at the probe trial 1, and (**E**) time spent around the target hole at the probe trial 2 in the good learners of mutants and wild-type controls.

Mutant mice of several strains showed lower performance during the training sessions, which might result in lower levels of recent and remote memory. In other words, the decreased performance in the remote memory test could simply reflect poor initial learning, instead of remote memory deficit per se. In fact, there were negative correlations between the number of errors in the last training session and the time spent around the target hole in the probe trial 1 (Figure 6A: for wild-type, *r* = −0.26, *P* =.0136; for mutants, *r* = −0.37, *P* =.0061) and probe trial 2 (Figure 6B: for wild-type, *r* = −0.34, *P* =.0008; for mutants, *r* = −0.27, *P* =.0488). To examine the possibility, we conducted a sensitivity analysis in which recent and remote memory performance was compared between good learners of mutant and wild-type mice with the number of errors in the last training session less than 2.92, the mean value obtained from wild-type mice. While there were no significant differences in the number of errors (Figure 6C: t_79_ = 1.18, *P* =.2402) and the time spent around the target hole in the first probe trial (Figure 6D: t_79_ = 0.77, *P* = .4426) between good learners of mutants and wild-type control mice, the mutants showed significantly less time spent around the target hole in the probe trial 2 compared to wild-type mice (Figure 6E: t_79_ = 3.39, *P* = .0011). Similar results were found in the cases of good learners showing shorter latency to reach the target hole or shorter distance traveled to reach the target hole (Figure S6). These data indicate that even good learners of mutant mice show poor performance in the remote memory test and support the idea that mutant mice exhibit pronounced remote memory deficits, even when they are successful in their initial learning.

### Cell Proliferation in the DG in Mutant Mice with iDG Phenotype

**Figure 7:**
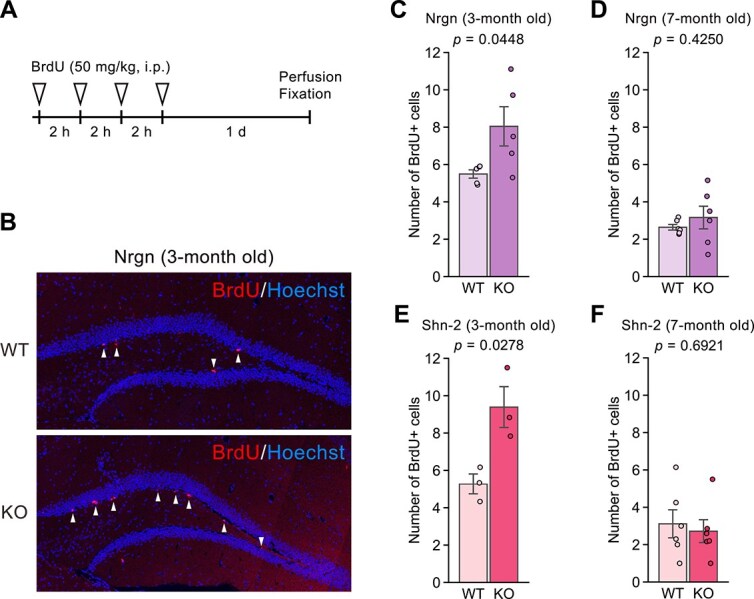
BrdU-positive cells in the hippocampal dentate gyrus in mutant mice with immature dentate gyrus phenotype. (**A**) Experimental procedure for BrdU labelling in the dentate gyrus in Nrgn KO, Shn-2 KO, and their age-matched WT control mice. (**B**) Representative images of BrdU-positive cells (white arrowheads) in the granular layer of the dentate gyrus in Nrgn KO and their WT mice. (**C**–**F**) the number of BrdU-positive cells in the dentate gyrus granular layer: (**C**) 3-month old Nrgn KO (n = 5) and WT (n = 5) mice, (**D**) 7-month old Nrgn KO (n = 6) and WT (n = 6) mice, (**E**) 3-month old Shn-2 KO (n = 3) and WT (n = 3) mice, (**F**) 7-month old Shn-2 KO (n = 6) and WT (n = 6) mice.

BrdU-positive cells in the granular zone of the DG were counted to assess cell proliferation and neurogenesis in Nrgn KO and Shn-2 KO mice during adulthood (Figure 7A–F). An increased number of BrdU-positive cells was found in 3-month-old Nrgn KO (Figure 7C: vs. WT, t_8_ = 2.38, *P*=.0448) and 3-month-old Shn-2 KO mice (Figure 7E: vs. WT, t_4_ = 3.38, *P*=.0278) compared to age-matched WT mice (see Table 2). Additional examinations showed that there were no significant differences in the number of BrdU-positive cells at the age of more than 7 months between Nrgn KO and their age-matched WT mice (Figure 7D: t_10_ = 0.83, *P*=.4250) and between Shn-2 KO and their age-matched WT mice (Figure 7F: t_10_ = 0.41, *P*=.6921). These data indicate increased neurogenesis in the granular zone of the DG in younger Nrgn KO and Shn-2 KO mice.

## DISCUSSION

The present study investigated the relationship between pseudo-immature states in the granule cells of the dentate gyrus, immature DG (iDG), and memory function by measuring recent and remote spatial reference memory in five mutant mouse models of neuropsychiatric and neurodegenerative disorders with the iDG phenotype, including Camk2a HET KO, Cn cKO, Nrgn KO, Shn-2 KO, and hAPP-J20 mice, in the Barnes circular maze task. The main findings are as follows: First, during the training sessions, all mutants showed improvement in spatial learning across the training sessions and were able to learn to selectively search and find the spatial location of the target, although decreased performance was observed in Camk2a HET KO, Cn cKO, and Nrgn KO mice. Second, in the probe test one day after the training session, Cn cKO, Nrgn KO, and Shn-2 KO mice showed normal spatial memory, as indicated by the lack of a significant difference in time spent around the target hole from WT mice, whereas Camk2a HET KO and J20 mice exhibited a shorter time spent around the target hole than WT mice, suggesting impaired recent memory. Third, all groups of mutant mice displayed decreased time spent around the target hole compared to WT mice in the probe test conducted four weeks after training, indicating that remote memory was impaired in all mutant groups. These findings demonstrate that remote spatial memory deficits are a commonly observed phenotype among mutant strains of mice with the iDG phenotype, suggesting that pseudo-immature states in the granule cells of the DG are involved in the retention and/or retrieval of remote memory.

Previous studies have reported that longer escape latencies were observed during training sessions in the Morris water maze task, another hippocampal-dependent task for spatial reference learning and memory, in Camk2a mutant mice,^95^ Cn cKO mice,^76^ Nrgn KO mice,^37^^,^^66^^,^^79^ and J20 mice.^30^^,^^80^^,^^82^^,^^99^^,^^100^ Consistent with these findings, our data revealed that Camk2a HET KO, Cn cKO, and Nrgn KO mice showed similar lower maze performance during training in the Barnes maze task, although J20 mice exhibited no significant differences in the maze performance compared to WT mice. Shn-2 KO mice exhibited slightly lower maze performance during training. Together, these results from the two types of maze tasks suggest that spatial learning during training was impaired in the mutants. However, it should be noted that deficits in maze performance might be due to their increased propensity to explore the maze and/or a decreased tendency to escape into the target hole, as suggested by the higher number of omission errors as well as hyperlocomotor activity and altered anxiety-like behavior in Camk2a HET KO mice,^19^ Cn cKO mice,^65^ Nrgn KO mice,^67^^,^^79^ and Shn-2 KO mice.^25^

The results of the probe test conducted one day after the last training session indicated that all groups of mutant mice showed no obvious differences in maze performance in the error counts, the latency, or the distance to first reach the target first reach the target hole compared to WT mice, which is suggestive of no deficits in motivational aspects to search for the target and spatial discrimination after training. The observation that Cn cKO, Nrgn KO, and Shn-2 KO mice spent a similar amount of time around the target location in the probe trial as their WT mice indicate that the mutants had intact recent spatial memory in the Barnes maze task, which is consistent with previous findings from the water maze test in Cn cKO mice^76^ and the Barnes maze test in Nrgn KO mice.^66^ Camk2a HET KO mice exhibited normal recent spatial memory when assessed in the Morris water maze task, with short retention delays of 3 days.^83^ Water-maze training is considered more stressful than Barnes maze training,^87^ possibly increasing the motivation to escape from the testing environment, which might contribute to their normal maze performance in the mutants. In the Barnes maze test, although our current study found that Camk2a HET KO and J20 mice showed recent memory deficits, it is possible that the decreased time spent around the target hole resulted from their hyperactive phenotype, increased motivation to explore the maze, or decreased escape tendency.

Deficits in remote memory have been reported in Camk2a HET KO mice when assessed in the Morris water maze task and fear conditioning paradigm, with longer retention delays of 10 days or more.^83^^,^^101^ Similarly, in this study, decreased performance in the remote memory test was found in Camk2a HET KO, Cn cKO, Nrgn KO, Shn-2 KO, and J20 mice in the Barnes maze task when assessed four weeks after training. It seems unlikely that their poor performance in the memory test could simply reflect poor initial learning, instead of remote memory deficit per se, because the decreased performance in the remote memory test was observed even in good learners of those mutant strains. These findings support the notion that the five mutant strains of mice exhibit remote memory deficits and that the iDG phenotype is associated with memory forgetting. Several hypotheses can be proposed to explain the remote memory deficits in the mutants. One of these is the morphological and functional synaptic abnormalities in mutants with the iDG phenotype. Altered granule cell excitability and reduced synaptic transmission in the DG, as seen in immature neurons,^21-24^ have been observed in Camk2a HET KO, Shn-2 KO, and J20 mice.^19^^,^^25^^,^^102^ The altered synaptic functions may be due to the immature dendritic spine morphology found in CKII HET KO mice^19^ and Shn-2 KO mice.^25^ Microglia and astrocytes play important roles in regulating synaptic elimination and NMDA-dependent long-term depression via postsynaptic GluA2 AMPAR endocytosis, thereby promoting forgetting.^103^^–^^105^ While the insertion of GluA2-containing AMPARs into post-synaptic sites is associated with synaptic strengthening and memory persistence, the removal of GluA2 AMPARs promotes synaptic depression and forgetting.^106^^–^^108^ The expression of complement C1q, which contributes to synaptic elimination,^109^^,^^110^ is increased in the brains of Shn-2 KO mice.^25^ Shn-2 KO, Cn cKO, and J20 mice exhibit increased expression of the astrocyte marker GFAP in the DG or CA1/3.^25^^,^^29^^,^^111^ Expression of GluA1 and GluA2 in DG granule cells, considered to be mature neuron makers, was decreased in Camk2a HET KO mice,^19^^,^^112^ Shn-2 KO mice,^26^ and Cn cKO mice.^29^ These findings suggest that altered synaptic organization may lead to the disruption of accessibility to previously stored memory at later time points in mutants with the iDG phenotype.

Accelerated forgetting of memories acquired during infancy, referred to as infantile amnesia, has been observed in rodents.^7^ During infancy, enhanced hippocampal neurogenesis occurs, which has been postulated to facilitate remodeling of hippocampal circuits through the integration of new neurons into existing circuits, resulting in the forgetting of previously acquired memory.^7^^,^^10^^–^^12^^,^^113^ The present and previous studies have shown that all groups of mutant mice exhibit an increased number of BrdU-positive cells in the DG at approximately 2–4 months of age,^19^^,^^20^^,^^92^^,^^93^ indicating enhanced neurogenesis at younger ages. The rate of neurogenesis declines with age.^114^^–^^116^ Our data showed that the number of BrdU-positive cells in 7-month-old Shn-2 KO and Nrgn KO mice was similar to that in the age-matched WT mice. A previous study reported that J20 mice displayed a similar level of neurogenesis as WT mice at 5–11 months of age.^93^ Considering that remote memory was assessed at approximately 4–11 months of age in this study, the involvement of neurogenesis in forgetting via continuous remodeling of the existing hippocampal circuits was expected to diminish in these mutants at the remote memory test, and the overlapping processes between adult neurogenesis and dematuration of granule cells in the DG could promote neuronal plasticity contributing to memory instability.

DG granule cells in all the mutants examined in this study show immaturity-related signatures, as assessed by the expression of molecular markers of neuronal maturation.^20^^,^^30^ The gene and protein expression levels of calbindin, a typical mature marker of DG granule cells, were decreased in the DG of the mutant mice.^19^^,^^25^^,^^27^^,^^29^ Several non-genetic factors have been suggested to postnatally induce immature status of DG granule cells, which is the phenomenon called as “dematuration.”^90^^,^^117^^,^^118^ For example, chronic administration of selective serotonin reuptake inhibitor, antidepressant fluoxetine (FLX), resulted in decreased calbindin expression in DG granule cells in adult mice.^90^ Calbindin is critical for hippocampal synaptic function and spatial learning and memory.^119^^–^^122^ Our previous study showed that chronic FLX treatment causes remote memory deficits.^7^ Increasing DG calbindin levels improved spatial object location memory in J20 mice.^122^ Based on these findings, calbindin expression within the DG may be a potentially critical factor contributing to memory deficits observed in mutant mice exhibiting the iDG phenotype. While memories may initially depend on the hippocampus, they become dependent on the cortex for their expression over time.^123^^,^^124^ Thus, remote memory deficits may be partially due to cortical neuronal abnormalities in mutant mice with the iDG phenotype. Camk2a HET KO mice showed decreased long-term potentiation in the cortex.^125^ Cn cKO mice exhibited impaired synaptic transmission and decreased high-frequency oscillatory activity in the prefrontal cortex.^126^ Chronic FLX administration reinstates cortical plasticity^127^ and induces dematuration of cortical neurons, as suggested by the increased expression of immature neuronal makers (PSA-NCAM) and decreased expression of markers of mature fast-spiking interneurons (parvalbumin-positive interneurons surrounded by perineuronal nets).^128^^–^^130^ Reduced Parvalbumin-positive cells in the medial prefrontal cortex have also been observed in Shn-2 KO mice.^25^ Cortical parvalbumin-positive interneurons and perineuronal nets have been reported to contribute to consolidation and retrieval of remote memory.^131^^,^^132^ Additional research is necessary to elucidate the role of neuronal abnormalities in various cell types across different brain regions, including DG granule cells, in remote memory formation within these mutant mice.

Genetic studies have revealed associations between mutations and dysregulated expression of CAMK2A gene and ID, epilepsy, ASD, and AD,^38^^–^^41^ between PPP3CA/PPP3CC gene encoding the Cn α-/γ-catalytic subunit and schizophrenia, ID, developmental delay, epilepsy, and autistic features,^42-52^ between NRGN gene and ID and developmental delay,^53^^–^^56^ between HIVEP2 gene and schizophrenia and ID,^59^^,^^60^ and between APP gene and AD.^62^^,^^63^ Cognitive dysfunction is a common feature of both neuropsychiatric and neurodegenerative disorders. Our previous studies and other reports have showed that behavioral abnormalities related to neuropsychiatric and neurodegenerative disorders, such as hyperlocomotor activity and working memory deficits, are commonly observed in Camk2a HET KO,^19^ Cn cKO,^29^^,^^65^ Nrgn KO,^66^^,^^67^ Shn-2 KO,^25^ and J20 mice,^133^ indicating that these mutants are animal models of neuropsychiatric and neurodegenerative disorders with good face and construct validity. Remote memory deficits have been documented in patients with schizophrenia^134^^,^^135^ and AD.^136^^–^^138^ The current study indicate that remote memory deficits are also a common behavioral phenotype in these mouse models.

In conclusion, the present study suggests that the iDG phenotype, the pseudo-immature state of DG granule cells, does not interfere with spatial learning and memory formation, but hinders retention/retrieval of remote spatial memory in certain mouse models of neuropsychiatric and neurodegenerative disorders. However, this study has some limitations that need to be addressed. First, iDG phenotype could be caused by an increase in adult neurogenesis, prolonged maturation time window of adult born granule cells, or “dematuration” of developmentally born granule cells during adulthood, either of which could lead to alteration of synaptic plasticity and/or stability of memory engram. Further investigation is needed to understand the underlying process of iDG phenotype and the primary cause of memory deficits in each mutant strain of mice. Second, our study does not exclude the possibility of the involvement of molecular and cellular abnormalities in brain regions and cell types other than DG granule cells, such as neuronal hyperexcitability, chronic inflammation, astrocyte activation, decreased brain pH, and altered metabolic changes, found in mutants with the iDG phenotype.^19^^,^^20^^,^^25^^,^^29^^,^^64^^,^^139^  Thus, although further studies are required to determine the extent of the contribution of the iDG phenotype to remote memory deficits by the development and application of a technique for the selective manipulation of the maturation state of DG granule cells, these mutant mouse models with iDG phenotype provides important clues to understand the pathophysiological mechanisms underlying remote memory deficits in neuropsychiatric and neurodegenerative disorders.

## Supplementary Material

Figure_S1_pyaf062

Figure_S2_pyaf062

Figure_S3_pyaf062

Figure_S4_pyaf062

Figure_S5_pyaf062

Figure_S6_pyaf062

Supplementary_Table_pyaf062

Supplementary_Table_R1_pyaf062

Supplementary_Captions_pyaf062

Supplementary_Text_pyaf062

## Data Availability

All the data used in this study are available from the authors upon request.

## References

[1] Eriksson PS, Perfilieva E, Björk-Eriksson T, et al. Neurogenesis in the adult human hippocampus. *Nat Med*. 1998;4:1313-1317. 10.1038/3305 9809557

[2] Kempermann G, Gast D, Kronenberg G, Yamaguchi M, Gage FH. Early determination and long-term persistence of adult-generated new neurons in the hippocampus of mice. *Development.* 2003;130:391-399. 10.1242/dev.00203 12466205

[3] Ngwenya LB, Heyworth NC, Shwe Y, Moore TL, Rosene DL. Age-related changes in dentate gyrus cell numbers, neurogenesis, and associations with cognitive impairments in the rhesus monkey. *Front Syst Neurosci*. 2015;9:102. 10.3389/fnsys.2015.00102 26236203 PMC4500920

[4] Clelland CD, Choi M, Romberg C, et al. A functional role for adult hippocampal neurogenesis in spatial pattern separation. *Science.* 2009;325:210-213. 10.1126/science.1173215 19590004 PMC2997634

[5] Burghardt NS, Park EH, Hen R, Fenton AA. Adult-born hippocampal neurons promote cognitive flexibility in mice. *Hippocampus.* 2012;22:1795-1808. 10.1002/hipo.22013 22431384 PMC4784987

[6] Nakashiba T, Cushman JD, Pelkey KA, et al. Young dentate granule cells mediate pattern separation, whereas old granule cells facilitate pattern completion. *Cell.* 2012;149:188-201. 10.1016/j.cell.2012.01.046 22365813 PMC3319279

[7] Akers KG, Martinez-Canabal A, Restivo L, et al. Hippocampal neurogenesis regulates forgetting during adulthood and infancy. *Science.* 2014;344:598-602. 10.1126/science.1248903 24812394

[8] Kitamura T, Inokuchi K. Role of adult neurogenesis in hippocampal-cortical memory consolidation. *Mol Brain.* 2014;7:13-18. 10.1186/1756-6606-7-13 24552281 PMC3942778

[9] McAvoy K, Besnard A, Sahay A. Adult hippocampal neurogenesis and pattern separation in DG: a role for feedback inhibition in modulating sparseness to govern population-based coding. *Front Syst Neurosci*. 2015;9:120. 10.3389/fnsys.2015.00120 26347621 PMC4542503

[10] Epp JR, Silva Mera R, Köhler S, Josselyn SA, Frankland PW. Neurogenesis-mediated forgetting minimizes proactive interference. *Nat Commun*. 2016;7:10838. 10.1038/ncomms10838 26917323 PMC4773435

[11] Ishikawa R, Fukushima H, Frankland PW, Kida S. Hippocampal neurogenesis enhancers promote forgetting of remote fear memory after hippocampal reactivation by retrieval. *Elife.* 2016;5:e17464. 10.7554/eLife.17464 27669409 PMC5036964

[12] Gao A, Xia F, Guskjolen AJ, et al. Elevation of hippocampal neurogenesis induces a temporally graded pattern of forgetting of contextual fear memories. *J Neurosci*. 2018;38:3190-3198. 10.1523/JNEUROSCI.3126-17.2018 29453206 PMC6596062

[13] Cuartero MI, de la Parra J, Pérez-Ruiz A, et al. Abolition of aberrant neurogenesis ameliorates cognitive impairment after stroke in mice. *J Clin Invest*. 2019;129:1536-1550. 10.1172/JCI120412 30676325 PMC6436875

[14] Tran LM, Josselyn SA, Richards BA, Frankland PW. Forgetting at biologically realistic levels of neurogenesis in a large-scale hippocampal model. *Behav Brain Res*. 2019;376:112180. 10.1016/j.bbr.2019.112180 31472193 PMC8719326

[15] Epp JR, Botly LC, Josselyn SA, Frankland PW. Voluntary exercise increases neurogenesis and mediates forgetting of complex paired associates memories. *Neuroscience.* 2021;475:1-9. 10.1016/j.neuroscience.2021.08.022 34464663 PMC8682805

[16] Ko SY, Frankland PW. Neurogenesis-dependent transformation of hippocampal engrams. *Neurosci Lett*. 2021;762:136176. 10.1016/j.neulet.2021.136176 34400284

[17] Scott GA, Terstege DJ, Roebuck AJ, et al. Adult neurogenesis mediates forgetting of multiple types of memory in the rat. *Mol Brain.* 2021;14:97. 10.1186/s13041-021-00808-4 34174906 PMC8236170

[18] Fujikawa R, Ramsaran AI, Guskjolen A, et al. Neurogenesis-dependent remodeling of hippocampal circuits reduces PTSD-like behaviors in adult mice. *Mol Psychiatry*. 2024;29:3316-3329. 10.1038/s41380-024-02585-7 38719894

[19] Yamasaki N, Maekawa M, Kobayashi K, et al. Alpha-CaMKII deficiency causes immature dentate gyrus, a novel candidate endophenotype of psychiatric disorders. *Mol Brain*. 2008;1:1-21. 10.1186/1756-6606-1-6 18803808 PMC2562999

[20] Hagihara H, Takao K, Walton NM, Matsumoto M, Miyakawa T. Immature dentate gyrus: an endophenotype of neuropsychiatric disorders. *Neural Plast*. 2013;2013:1-24. 10.1155/2013/318596 PMC3694492 23840971

[21] Liu XS, Tilwalli S, Ye GL, Lio PA, Pasternak JF, Trommer BL. Morphologic and electrophysiologic maturation in developing dentate gyrus granule cells. *Brain Res*. 2000;856:202-212. 10.1016/S0006-8993(99)02421-X 10677627

[22] Ambrogini P, Lattanzi D, Ciuffoli S, et al. Morpho-functional characterization of neuronal cells at different stages of maturation in granule cell layer of adult rat dentate gyrus. *Brain Res*. 2004;1017:21-31. 10.1016/j.brainres.2004.05.039 15261095

[23] Marchal C, Mulle C. Postnatal maturation of mossy fibre excitatory transmission in mouse CA3 pyramidal cells: a potential role for kainate receptors. *J Physiol*. 2004;561:27-37. 10.1113/jphysiol.2004.069922 15358807 PMC1665334

[24] Schmidt-Hieber C, Jonas P, Bischofberger J. Enhanced synaptic plasticity in newly generated granule cells of the adult hippocampus. *Nature.* 2004;429:184-187. 10.1038/nature02553 15107864

[25] Takao K, Kobayashi K, Hagihara H, et al. Deficiency of schnurri-2, an MHC enhancer binding protein, induces mild chronic inflammation in the brain and confers molecular, neuronal, and behavioral phenotypes related to schizophrenia. *Neuropsychopharmacology.* 2013;38:1409-1425. 10.1038/npp.2013.38 23389689 PMC3682135

[26] Nakao A, Miyazaki N, Ohira K, et al. Immature morphological properties in subcellular-scale structures in the dentate gyrus of Schnurri-2 knockout mice: a model for schizophrenia and intellectual disability. *Mol Brain.* 2017;10:1-11. 10.1186/s13041-017-0339-2 29233179 PMC5727961

[27] Hattori S, Hagihara H, Huang FL, et al. Deficiency of neurogranin, a susceptible gene for schizophrenia, causes behavioral phenotypes related to schizophrenia and immaturity of the dentate gyrus in mice. *Int J Neuropsychopharmacol*. 2016;19:36-37. 10.1093/ijnp/pyw041.374

[28] Ohira K, Kobayashi K, Toyama K, et al. Synaptosomal-associated protein 25 mutation induces immaturity of the dentate granule cells of adult mice. *Mol Brain.* 2013;6:12. 10.1186/1756-6606-6-12 23497716 PMC3605216

[29] Hagihara H, Shoji H, Kuroiwa M, et al. Forebrain-specific conditional calcineurin deficiency induces dentate gyrus immaturity and hyper-dopaminergic signaling in mice. *Mol Brain*. 2022;15:94. 10.1186/s13041-022-00981-0 36414974 PMC9682671

[30] Palop JJ, Jones B, Kekonius L, et al. Neuronal depletion of calcium-dependent proteins in the dentate gyrus is tightly linked to Alzheimer's disease-related cognitive deficits. *Proc Natl Acad Sci USA*. 2003;100:9572-9577. 10.1073/pnas.1133381100 12881482 PMC170959

[31] Palop JJ, Chin J, Roberson ED, et al. Aberrant excitatory neuronal activity and compensatory remodeling of inhibitory hippocampal circuits in mouse models of Alzheimer's disease. *Neuron.* 2007;55:697-711. 10.1016/j.neuron.2007.07.025 17785178 PMC8055171

[32] Naganishi S, Hagihara H, Miyakawa T. Gene expression signatures of immaturity, decreased pH, and neural hyperexcitation in the hippocampus of Alzheimer's disease model mice. *Neuropsychopharmacol Rep*. 2025;45:e70001. 10.1002/npr2.70001 39907034 PMC11795175

[33] Nagahara AH, Merrill DA, Coppola G, et al. Neuroprotective effects of brain-derived neurotrophic factor in rodent and primate models of Alzheimer's disease. *Nat Med*. 2009;15:331-337. 10.1038/nm.1912 19198615 PMC2838375

[34] Lisman J, Schulman H, Cline H. The molecular basis of CaMKII function in synaptic and behavioural memory. *Nat Rev Neurosci*. 2002;3:175-190. 10.1038/nrn753 11994750

[35] Groth RD, Dunbar RL, Mermelstein PG. Calcineurin regulation of neuronal plasticity. *Biochem Biophys Res Commun*. 2003;311:1159-1171. 10.1016/j.bbrc.2003.09.002 14623302

[36] Yasuda R, Hayashi Y, Hell JW. CaMKII: a central molecular organizer of synaptic plasticity, learning and memory. *Nat Rev Neurosci*. 2022;23:666-682. 10.1038/s41583-022-00624-2 36056211

[37] Pak JH, Huang FL, Li J, et al. Involvement of neurogranin in the modulation of calcium/calmodulin-dependent protein kinase II, synaptic plasticity, and spatial learning: a study with knockout mice. *Proc Natl Acad Sci USA*. 2000;97:11232-11237. 10.1073/pnas.210184697 11016969 PMC17183

[38] Ament SA, Szelinger S, Glusman G, et al. Rare variants in neuronal excitability genes influence risk for bipolar disorder. *Proc Natl Acad Sci*. 2015;112:3576-3581. 10.1073/pnas.1424958112 25730879 PMC4371952

[39] Iossifov I, O’roak BJ, Sanders SJ, et al. The contribution of de novo coding mutations to autism spectrum disorder. *Nature.* 2014;515:216-221. 10.1038/nature13908 25363768 PMC4313871

[40] Ghosh A, Giese KP. Calcium/calmodulin-dependent kinase II and Alzheimer’s disease. *Mol Brain.* 2015;8:1-7. 10.1186/s13041-015-0166-2 26603284 PMC4657223

[41] Chia PH, Zhong FL, Niwa S, et al. A homozygous loss-of-function CAMK2A mutation causes growth delay, frequent seizures and severe intellectual disability. *Elife.* 2018;7:e32451. 10.7554/eLife.32451 29784083 PMC5963920

[42] Gerber DJ, Hall D, Miyakawa T, et al. Evidence for association of schizophrenia with genetic variation in the 8p21. 3 gene, PPP3CC, encoding the calcineurin gamma subunit. *Proc Natl Acad Sci USA*. 2003;100:8993-8998. 10.1073/pnas.1432927100 12851458 PMC166426

[43] Horiuchi Y, Ishiguro H, Koga M, et al. Support for association of the PPP3CC gene with schizophrenia. *Mol Psychiatry*. 2007;12:891-893. 10.1038/sj.mp.4002019 17895921

[44] Liu YL, Fann CSJ, Liu CM, et al. More evidence supports the association of PPP3CC with schizophrenia. *Mol Psychiatry*. 2007;12:966-974. 10.1038/sj.mp.4001977 17339875

[45] Yamada K, Gerber DJ, Iwayama Y, et al. Genetic analysis of the calcineurin pathway identifies members of the EGR gene family, specifically EGR3, as potential susceptibility candidates in schizophrenia. *Proc Natl Acad Sci USA*. 2007;104:2815-2820. 10.1073/pnas.0610765104 17360599 PMC1815264

[46] Singh T, Poterba T, Curtis D, et al. Rare coding variants in ten genes confer substantial risk for schizophrenia. *Nature.* 2022;604:509-516. 10.1038/s41586-022-04556-w 35396579 PMC9805802

[47] Myers CT, Stong N, Mountier EI, et al. De novo mutations in PPP3CA cause severe neurodevelopmental disease with seizures. *Am J Hum Genet*. 2017;101:516-524. 10.1016/j.ajhg.2017.08.013 28942967 PMC5630160

[48] Mizuguchi T, Nakashima M, Kato M, et al. Loss-of-function and gain-of-function mutations in PPP3CA cause two distinct disorders. *Hum Mol Genet*. 2018;27:1421-1433. 10.1093/hmg/ddy052 29432562

[49] Qian Y, Wu B, Lu Y, et al. Early-onset infant epileptic encephalopathy associated with a de novo PPP3CA gene mutation. *Mol Case Stud*. 2018;4:a002949. 10.1101/mcs.a002949 PMC6318765 30455226

[50] Li J, Gao K, Yan H, et al. Reanalysis of whole exome sequencing data in patients with epilepsy and intellectual disability/mental retardation. *Gene.* 2019;700:168-175. 10.1016/j.gene.2019.03.037 30904718

[51] Rydzanicz M, Wachowska M, Cook EC, et al. Novel calcineurin a (PPP3CA) variant associated with epilepsy, constitutive enzyme activation and downregulation of protein expression. *Eur J Hum Genet*. 2019;27:61-69. 10.1038/s41431-018-0254-8 30254215 PMC6303256

[52] Panneerselvam S, Wang J, Zhu W, et al. PPP3CA truncating variants clustered in the regulatory domain cause early-onset refractory epilepsy. *Clin Genet*. 2021;100:227-233. 10.1111/cge.13979 33963760 PMC11698261

[53] Ruano D, Aulchenko YS, Macedo A, et al. Association of the gene encoding neurogranin with schizophrenia in males. *J Psychiatr Res*. 2008;42:125-133. 10.1016/j.jpsychires.2006.10.008 17140601

[54] Stefansson H, Ophoff RA, Steinberg S, et al. Common variants conferring risk of schizophrenia. *Nature.* 2009;460:744-747. 10.1038/nature08186 19571808 PMC3077530

[55] Shen YC, Tsai HM, Cheng MC, Hsu SH, Chen SF, Chen CH. Genetic and functional analysis of the gene encoding neurogranin in schizophrenia. *Schizophr Res*. 2012;137:7-13. 10.1016/j.schres.2012.01.011 22306195

[56] Coldren CD, Lai Z, Shragg P, et al. Chromosomal microarray mapping suggests a role for BSX and neurogranin in neurocognitive and behavioral defects in the 11q terminal deletion disorder (Jacobsen syndrome). *Neurogenetics.* 2009;10:89-95. 10.1007/s10048-008-0157-x 18855024 PMC3050515

[57] Dörflinger U, Pscherer A, Moser M, Rümmele P, Schüle R, Buettner R. Activation of somatostatin receptor II expression by transcription factors MIBP1 and SEF-2 in the murine brain. *Mol Cell Biol*. 1999;19:3736-3747. 10.1128/MCB.19.5.3736 10207097 PMC84194

[58] Takagi T, Harada J, Ishii S. Murine Schnurri-2 is required for positive selection of thymocytes. *Nat Immunol*. 2001;2:1048-1053. 10.1038/ni728 11668343

[59] Srivastava S, Engels H, Schanze I, et al. Loss-of-function variants in HIVEP2 are a cause of intellectual disability. *Eur J Hum Genet*. 2016;24:556-561. 10.1038/ejhg.2015.151 26153216 PMC4929870

[60] Steinfeld H, Cho MT, Retterer K, et al. Mutations in HIVEP2 are associated with developmental delay, intellectual disability, and dysmorphic features. *Neurogenetics.* 2016;17:159-164. 10.1007/s10048-016-0479-z 27003583 PMC4907844

[61] Tyan SH, Shih AYJ, Walsh JJ, et al. Amyloid precursor protein (APP) regulates synaptic structure and function. *Mol Cell Neurosci*. 2012;51:43-52. 10.1016/j.mcn.2012.07.009 22884903 PMC3538857

[62] Thinakaran G, Koo EH. Amyloid precursor protein trafficking, processing, and function. *J Biol Chem*. 2008;283:29615-29619. 10.1074/jbc.R800019200 18650430 PMC2573065

[63] O'brien RJ, Wong PC. Amyloid precursor protein processing and Alzheimer's disease. Ann. *Rev Neurosci*. 2011;34:185-204. 10.1146/annurev-neuro-061010-113613 PMC3174086 21456963

[64] Hagihara H, Horikawa T, Nakamura HK, et al. Circadian gene circuitry predicts hyperactive behavior in a mood disorder mouse model. *Cell Rep*. 2016;14:2784-2796. 10.1016/j.celrep.2016.02.067 27028761

[65] Miyakawa T, Leiter LM, Gerber DJ, et al. Conditional calcineurin knockout mice exhibit multiple abnormal behaviors related to schizophrenia. *Proc Natl Acad Sci USA*. 2003;100:8987-8992. 10.1073/pnas.1432926100 12851457 PMC166425

[66] Miyakawa T, Yared E, Pak JH, Huang FL, Huang KP, Crawley JN. Neurogranin null mutant mice display performance deficits on spatial learning tasks with anxiety related components. *Hippocampus.* 2001;11:763-775. 10.1002/hipo.1092 11811671

[67] Nakajima R, Hattori S, Funasaka T, Huang FL, Miyakawa T. Decreased nesting behavior, selective increases in locomotor activity in a novel environment, and paradoxically increased open arm exploration in neurogranin knockout mice. *Neuropsychopharmacol Rep*. 2021;41:111-116. 10.1002/npr2.12150 33270377 PMC8182962

[68] Lanooij SD, Eisel UL, van der Zee EA, Kas MJ. Variation in group composition alters an early-stage social phenotype in hAPP-transgenic J20 mice. J Alzheimer’s Dis. 2023;93:211-224. 10.3233/JAD-221126 36970900 PMC10200156

[69] Oroszi T, Geerts E, Rajadhyaksha R, Nyakas C, van Heuvelen MJ, van der Zee EA. Whole-body vibration ameliorates glial pathological changes in the hippocampus of hAPP transgenic mice, but does not affect plaque load. Behav Brain Funct. 2023;19:5. 10.1186/s12993-023-00208-9 PMC10026461 36941713

[70] Quartey MO, Nyarko JN, Pennington PR, et al. Age-and sex-dependent profiles of APP fragments and key secretases align with changes in despair-like behavior and cognition in young APPSwe/Ind mice. Biochem Biophys Res Commun. 2019;511:454-459. 10.1016/j.bbrc.2019.02.083 30803762

[71] Esposito L, Raber J, Kekonius L, et al. Reduction in mitochondrial superoxide dismutase modulates Alzheimer's disease-like pathology and accelerates the onset of behavioral changes in human amyloid precursor protein transgenic mice. J Neurosci. 2006;26:5167-5179. 10.1523/JNEUROSCI.0482-06.2006 16687508 PMC6674260

[72] Gandal MJ, Nesbitt AM, McCurdy RM, Alter MD. Measuring the maturity of the fast-spiking interneuron transcriptional program in autism, schizophrenia, and bipolar disorder. *PLoS One*. 2012;7:e41215. 10.1371/journal.pone.0041215 22936973 PMC3427326

[73] Walton NM, Zhou Y, Kogan JH, et al. Detection of an immature dentate gyrus feature in human schizophrenia/bipolar patients. *Transl Psychiatry*. 2012;2:e135. 10.1038/tp.2012.56 22781168 PMC3410619

[74] Hagihara H, Ohira K, Takao K, Miyakawa T. Transcriptomic evidence for immaturity of the prefrontal cortex in patients with schizophrenia. *Mol Brain.* 2014;7:1-18. 10.1186/1756-6606-7-41 24886351 PMC4066280

[75] Murano T, Hagihara H, Tajinda K, Matsumoto M, Miyakawa T. Transcriptomic immaturity inducible by neural hyperexcitation is shared by multiple neuropsychiatric disorders. *Commun Biol*. 2019;2:1-11. 10.1038/s42003-018-0277-2 30675529 PMC6342824

[76] Zeng H, Chattarji S, Barbarosie M, et al. Forebrain-specific calcineurin knockout selectively impairs bidirectional synaptic plasticity and working/episodic-like memory. *Cell.* 2001;107:617-629. 10.1016/S0092-8674(01)00585-2 11733061

[77] Shoji H, Hagihara H, Takao K, Hattori S, Miyakawa T. T-maze forced alternation and left-right discrimination tasks for assessing working and reference memory in mice. *J Vis Exp*. 2012;60:e3300. 10.3791/3300 PMC3399492 22395674

[78] Huang FL, Huang KP, Wu J, Boucheron C. Environmental enrichment enhances neurogranin expression and hippocampal learning and memory but fails to rescue the impairments of neurogranin null mutant mice. *J Neurosci*. 2006;26:6230-6237. 10.1523/JNEUROSCI.1182-06.2006 16763030 PMC6675199

[79] Huang FL, Huang KP. Methylphenidate improves the behavioral and cognitive deficits of neurogranin knockout mice. *Genes Brain Behav*. 2012;11:794-805. 10.1111/j.1601-183X.2012.00825.x 22809330 PMC3467336

[80] Galvan V, Gorostiza OF, Banwait S, et al. Reversal of Alzheimer's-like pathology and behavior in human APP transgenic mice by mutation of Asp664. *Proc Natl Acad Sci USA*. 2006;103:7130-7135. 10.1073/pnas.0509695103 16641106 PMC1459029

[81] Cheng IH, Scearce-Levie K, Legleiter J, et al. Accelerating amyloid-β fibrillization reduces oligomer levels and functional deficits in Alzheimer disease mouse models. *J Biol Chem*. 2007;282:23818-23828. 10.1074/jbc.M701078200 17548355

[82] Cissé M, Sanchez PE, Kim DH, Ho K, Yu GQ, Mucke L. Ablation of cellular prion protein does not ameliorate abnormal neural network activity or cognitive dysfunction in the J20 line of human amyloid precursor protein transgenic mice. *J Neurosci*. 2011;31:10427-10431. 10.1523/JNEUROSCI.1459-11.2011 21775587 PMC3314063

[83] Frankland PW, O'Brien C, Ohno M, Kirkwood A, Silva AJ. Α-CaMKII-dependent plasticity in the cortex is required for permanent memory. *Nature.* 2001;411:309-313. 10.1038/35077089 11357133

[84] Barnes CA . Memory deficits associated with senescence: a neurophysiological and behavioral study in the rat. *J Comp Physiol Psychol*. 1979;93:74-104. 10.1037/h0077579 221551

[85] Kennard JA, Woodruff-Pak DS. Age sensitivity of behavioral tests and brain substrates of normal aging in mice. *Front Aging Neurosci*. 2011;3:9. 10.3389/fnagi.2011.00009 21647305 PMC3103996

[86] Gawel K, Gibula E, Marszalek-Grabska M, Filarowska J, Kotlinska JH. Assessment of spatial learning and memory in the Barnes maze task in rodents—methodological consideration. *N-S Arch Pharmacol*. 2019;392:1-18. 10.1007/s00210-018-1589-y PMC6311199 30470917

[87] Harrison FE, Hosseini AH, McDonald MP. Endogenous anxiety and stress responses in water maze and Barnes maze spatial memory tasks. *Behav Brain Res*. 2009;198:247-251. 10.1016/j.bbr.2008.10.015 18996418 PMC2663577

[88] Wolfer DP, Stagljar-Bozicevic M, Errington ML, Lipp HP. Spatial memory and learning in transgenic mice: fact or artifact? *Physiology.* 1998;13:118-123. 10.1152/physiologyonline.1998.13.3.118 11390774

[89] Paul CM, Magda G, Abel S. Spatial memory: theoretical basis and comparative review on experimental methods in rodents. *Behav Brain Res*. 2009;203:151-164. 10.1016/j.bbr.2009.05.022 19467271

[90] Kobayashi K, Ikeda Y, Sakai A, et al. Reversal of hippocampal neuronal maturation by serotonergic antidepressants. *Proc Natl Acad Sci USA*. 2010;107:8434-8439. 10.1073/pnas.0912690107 20404165 PMC2889553

[91] Malberg JE, Eisch AJ, Nestler EJ, Duman RS. Chronic antidepressant treatment increases neurogenesis in adult rat hippocampus. *J Neurosci*. 2000;20:9104-9110. 10.1523/JNEUROSCI.20-24-09104.2000 11124987 PMC6773038

[92] Jin K, Galvan V, Xie L, et al. Enhanced neurogenesis in Alzheimer's disease transgenic (PDGF-APPSw, Ind) mice. *Proc Natl Acad Sci USA*. 2004;101:13363-13367. 10.1073/pnas.0403678101 15340159 PMC516572

[93] López-Toledano MA, Shelanski ML. Increased neurogenesis in young transgenic mice overexpressing human APP(Sw, Ind). *J Alzheimer's Dis*. 2007;12:229-240. 10.3233/JAD-2007-12304 18057556

[94] Pan H, Wang D, Zhang X, et al. Amyloid β is not the major factor accounting for impaired adult hippocampal neurogenesis in mice overexpressing amyloid precursor protein. Stem Cell Rep. 2016;7:707-718. 10.1016/j.stemcr.2016.08.019 PMC5063569 27693425

[95] Silva AJ, Paylor R, Wehner JM, Tonegawa S. Impaired spatial learning in α-calcium-calmodulin kinase II mutant mice. *Science.* 1992;257:206-211. 10.1126/science.1321493 1321493

[96] Neilson JR, Winslow MM, Hur EM, Crabtree GR. Calcineurin B1 is essential for positive but not negative selection during thymocyte development. *Immunity.* 2004;20:255-266. 10.1016/S1074-7613(04)00052-4 15030770

[97] Tsien JZ, Huerta PT, Tonegawa S. The essential role of hippocampal CA1 NMDA receptor–dependent synaptic plasticity in spatial memory. *Cell.* 1996;87:1327-1338. 10.1016/S0092-8674(00)81827-9 8980238

[98] Mucke L, Masliah E, Yu GQ, et al. High-level neuronal expression of Aβ1–42 in wild-type human amyloid protein precursor transgenic mice: Synaptotoxicity without plaque formation. *J Neurosci*. 2000;20:4050-4058. 10.1523/JNEUROSCI.20-11-04050.2000 10818140 PMC6772621

[99] Murakami K, Yokoyama SI, Murata N, et al. Insulin receptor mutation results in insulin resistance and hyperinsulinemia but does not exacerbate Alzheimer’s-like phenotypes in mice. *Biochem Biophys Res Commun*. 2011;409:34-39. 10.1016/j.bbrc.2011.04.101 21549686

[100] Meilandt WJ, Yu GQ, Chin J, et al. Enkephalin elevations contribute to neuronal and behavioral impairments in a transgenic mouse model of Alzheimer's disease. *J Neurosci*. 2008;28:5007-5017. 10.1523/JNEUROSCI.0590-08.2008 18463254 PMC3315282

[101] Frankland PW, Bontempi B, Talton LE, Kaczmarek L, Silva AJ. The involvement of the anterior cingulate cortex in remote contextual fear memory. *Science.* 2004;304:881-883. 10.1126/science.1094804 15131309

[102] Zhang X, Wei X, Mei Y, et al. Modulating adult neurogenesis affects synaptic plasticity and cognitive functions in mouse models of Alzheimer's disease. *Stem Cell Rep*. 2021;16:3005-3019. 10.1016/j.stemcr.2021.11.003 PMC8693766 34861165

[103] Navarrete M, Cuartero MI, Palenzuela R, et al. Astrocytic p38α MAPK drives NMDA receptor-dependent long-term depression and modulates long-term memory. *Nat Commun*. 2019;10:2968. 10.1038/s41467-019-10830-9 31273206 PMC6609681

[104] Wang C, Yue H, Hu Z, et al. Microglia mediate forgetting via complement-dependent synaptic elimination. *Science.* 2020;367:688-694. 10.1126/science.aaz2288 32029629

[105] Lee JH, Kim JY, Noh S, et al. Astrocytes phagocytose adult hippocampal synapses for circuit homeostasis. *Nature.* 2021;590:612-617. 10.1038/s41586-020-03060-3 33361813

[106] Guskjolen AJ . Losing connections, losing memory: Ampa receptor endocytosis as a neurobiological mechanism of forgetting. *J Neurosci*. 2016;36:7559-7561. 10.1523/JNEUROSCI.1445-16.2016 27445134 PMC6705558

[107] Migues PV, Liu L, Archbold GE, et al. Blocking synaptic removal of GluA2-containing AMPA receptors prevents the natural forgetting of long-term memories. *J Neurosci*. 36:3481-3494. 27013677 10.1523/JNEUROSCI.3333-15.2016 PMC6601735

[108] Awasthi A, Ramachandran B, Ahmed S, et al. Synaptotagmin-3 drives AMPA receptor endocytosis, depression of synapse strength, and forgetting. *Science.* 2019;363:aav1483. 10.1126/science.aav1483 30545844

[109] Stevens B, Allen NJ, Vazquez LE, et al. The classical complement cascade mediates CNS synapse elimination. *Cell.* 2007;131:1164-1178. 10.1016/j.cell.2007.10.036 18083105

[110] Stephan AH, Barres BA, Stevens B. The complement system: an unexpected role in synaptic pruning during development and disease. *Ann Rev Neurosci*. 2012;35:369-389. 10.1146/annurev-neuro-061010-113810 22715882

[111] Wright AL, Zinn R, Hohensinn B, et al. Neuroinflammation and neuronal loss precede aβ plaque deposition in the hAPP-J20 mouse model of Alzheimer’s disease. *PLoS One*. 2013;8:e59586. 10.1371/journal.pone.0059586 23560052 PMC3613362

[112] Hagihara H, Ohira K, Toyama K, Miyakawa T. Expression of the AMPA receptor subunits GluR1 and GluR2 is associated with granule cell maturation in the dentate gyrus. *Front Neurosci*. 2011;5:100. 10.3389/fnins.2011.00100 21927594 PMC3168919

[113] Frankland PW, Köhler S, Josselyn SA. Hippocampal neurogenesis and forgetting. *Trends Neurosci*. 2013;36:497-503. 10.1016/j.tins.2013.05.002 23768770

[114] Seki T, Arai Y. Age-related production of new granule cells in the adult dentate gyrus. *Neuroreport.* 1995;6:2479-2482. 10.1097/00001756-199512150-00010 8741746

[115] Kuhn HG, Dickinson-Anson H, Gage FH. Neurogenesis in the dentate gyrus of the adult rat: age-related decrease of neuronal progenitor proliferation. *J Neurosci*. 1996;16:2027-2033. 10.1523/JNEUROSCI.16-06-02027.1996 8604047 PMC6578509

[116] Kempermann G, Gast D, Gage FH. Neuroplasticity in old age: sustained fivefold induction of hippocampal neurogenesis by long-term environmental enrichment. *Ann Neurol*. 2002;52:135-143. 10.1002/ana.10262 12210782

[117] Shin R, Kobayashi K, Hagihara H, et al. The immature dentate gyrus represents a shared phenotype of mouse models of epilepsy and psychiatric disease. *Bipolar Disord*. 2013;15:405-421. 10.1111/bdi.12064 23560889 PMC3752967

[118] Imoto Y, Segi-Nishida E, Suzuki H, Kobayashi K. Rapid and stable changes in maturation-related phenotypes of the adult hippocampal neurons by electroconvulsive treatment. *Mol Brain.* 2017;10:1-15. 10.1186/s13041-017-0288-9 28253930 PMC5335812

[119] Molinari S, Battini R, Ferrari S, et al. Deficits in memory and hippocampal long-term potentiation in mice with reduced calbindin D-28K expression. *Proc Natl Acad Sci USA*. 1996;93:8028-8033. 10.1073/pnas.93.15.8028 8755597 PMC38869

[120] Jouvenceau A, Potier B, Battini R, Ferrari S, Dutar P, Billard JM. Glutamatergic synaptic responses and long-term potentiation are impaired in the CA1 hippocampal area of calbindin D28k-deficient mice. *Synapse.* 1999;33:172-180. 10.1002/(SICI)1098-2396(19990901)33:3<172::AID-SYN2>3.0.CO;2-S 10420165

[121] Westerink RHS, Beekwilder JP, Wadman WJ. Differential alterations of synaptic plasticity in dentate gyrus and CA1 hippocampal area of calbindin-D28K knockout mice. *Brain Res*. 2012;1450:1-10. 10.1016/j.brainres.2012.02.036 22405690

[122] You JC, Muralidharan K, Park JW, et al. Epigenetic suppression of hippocampal calbindin-D28k by ΔFosB drives seizure-related cognitive deficits. *Nat Med*. 2017;23:1377-1383. 10.1038/nm.4413 29035369 PMC5747956

[123] Squire LR, Alvarez P. Retrograde amnesia and memory consolidation: a neurobiological perspective. *Curr Opin Neurobiol*. 1995;5:169-177. 10.1016/0959-4388(95)80023-9 7620304

[124] Frankland PW, Bontempi B. The organization of recent and remote memories. *Nat Rev Neurosci*. 2005;6:119-130. 10.1038/nrn1607 15685217

[125] Elgersma Y, Sweatt JD, Giese KP. Mouse genetic approaches to investigating calcium/calmodulin-dependent protein kinase II function in plasticity and cognition. *J Neurosci*. 2004;24:8410-8415. 10.1523/JNEUROSCI.3622-04.2004 15456813 PMC6729904

[126] Cottrell JR, Levenson JM, Kim SH, et al. Working memory impairment in calcineurin knock-out mice is associated with alterations in synaptic vesicle cycling and disruption of high-frequency synaptic and network activity in prefrontal cortex. *J Neurosci*. 2013;33:10938-10949. 10.1523/JNEUROSCI.5362-12.2013 23825400 PMC3718364

[127] Vetencourt JFM, Sale A, Viegi A, et al. The antidepressant fluoxetine restores plasticity in the adult visual cortex. *Science.* 2008;320:385-388. 10.1126/science.1150516 18420937

[128] Karpova NN, Pickenhagen A, Lindholm J, et al. Fear erasure in mice requires synergy between antidepressant drugs and extinction training. *Science.* 2011;334:1731-1734. 10.1126/science.1214592 22194582 PMC3929964

[129] Ohira K, Takeuchi R, Iwanaga T, Miyakawa T. Chronic fluoxetine treatment reduces parvalbumin expression and perineuronal netsin gamma-aminobutyric acidergic interneurons of the frontal cortex in adultmice. *Mol Brain.* 2013;6:43-11. 10.1186/1756-6606-6-43 24228616 PMC4225860

[130] Ohira K, Takeuchi R, Shoji H, Miyakawa T. Fluoxetine-induced cortical adult neurogenesis. *Neuropsychopharmacology.* 2013;38:909-920. 10.1038/npp.2013.2 23303069 PMC3629401

[131] Xia F, Richards BA, Tran MM, Josselyn SA, Takehara-Nishiuchi K, Frankland PW. Parvalbumin-positive interneurons mediate neocortical-hippocampal interactions that are necessary for memory consolidation. *Elife.* 2017;6:e27868. 10.7554/eLife.27868 28960176 PMC5655147

[132] Thompson EH, Lensjø KK, Wigestrand MB, Malthe-Sørenssen A, Hafting T, Fyhn M. Removal of perineuronal nets disrupts recall of a remote fear memory. *Proc Natl Acad Sci USA*. 2018;115:607-612. 10.1073/pnas.1713530115 29279411 PMC5776974

[133] Palop JJ, Mucke L. Network abnormalities and interneuron dysfunction in Alzheimer disease. *Nat Rev Neurosci*. 2016;17:777-792. 10.1038/nrn.2016.141 27829687 PMC8162106

[134] Tamlyn D, McKenna PJ, Mortimer AM, Lund CE, Hammond S, Baddeley AD. Memory impairment in schizophrenia: its extent, affiliations and neuropsychological character. *Psychol Med*. 1992;22:101-115. 10.1017/S0033291700032773 1349439

[135] Feinstein A, Goldberg TE, Nowlin B, Weinberger DR. Types and characteristics of remote memory impairment in schizophrenia. *Schizophr Res*. 1998;30:155-163. 10.1016/S0920-9964(97)00129-1 9549779

[136] Beatty WW, Salmon DP. Remote memory for visuospatial information in patients with Alzheimer's disease. *J Geriatr Psychiatry Neurol*. 1991;4:14-17. 10.1177/089198879100400103 2054046

[137] Dorrego MF, Sabe L, García Cuerva A, et al. Remote memory in Alzheimer's disease. *J Neuropsychiatry Clin Neurosci*. 1999;11:490-497. 10.1176/jnp.11.4.490 10570763

[138] Sartori G, Snitz BE, Sorcinelli L, Daum I. Remote memory in advanced Alzheimer’s disease. *Arch Clin Neuropsychol*. 2004;19:779-789. 10.1016/j.acn.2003.09.007 15288331

[139] Hagihara H, Catts VS, Katayama Y, et al. Decreased brain pH as a shared endophenotype of psychiatric disorders. *Neuropsychopharmacology.* 2018;43:459-468. 10.1038/npp.2017.167 28776581 PMC5770757

