# Supplementary material for: Remote spatial memory deficits in mouse models of neuropsychiatric disorders with immature dentate gyrus phenotype
Source: Int J Neuropsychopharmacol. 2025 Aug 23;28(10):pyaf062. doi: 10.1093/ijnp/pyaf062 (PMC12553137; doi:10.1093/ijnp/pyaf062)
Supplement: Figure_S1_pyaf062 [file figure_s1_pyaf062.pdf]

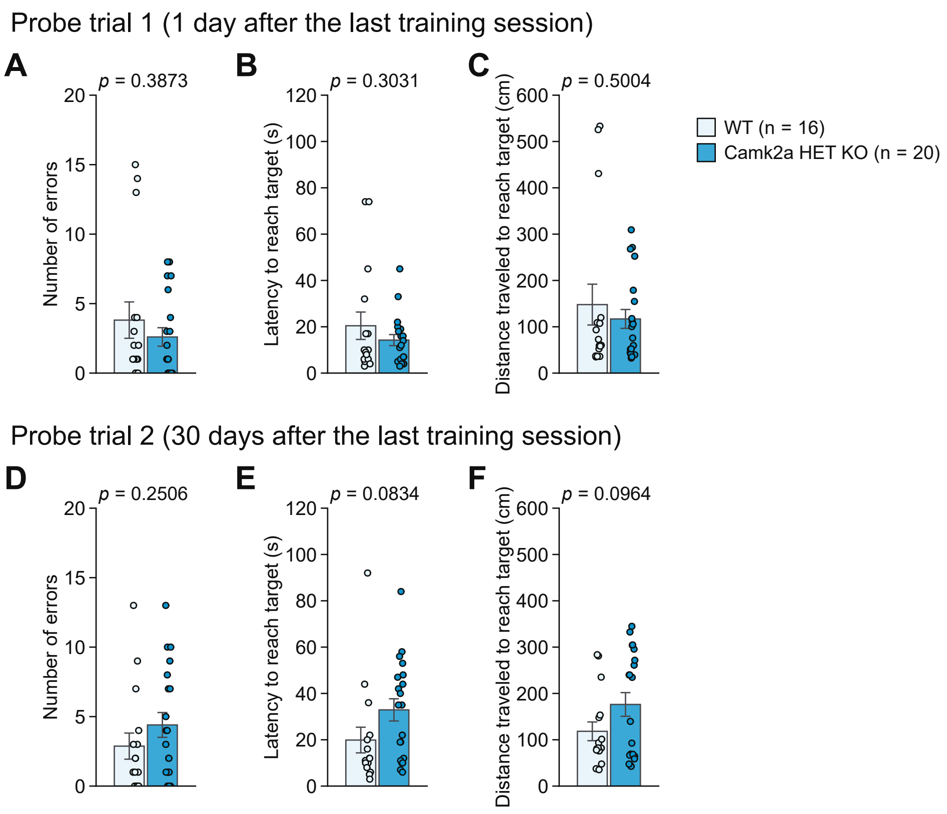

Probe trial 1 (1 day after the last training session)
A
p = 0.3873
Number of errors
B
p = 0.3031
Latency to reach target (s)
C
p = 0.5004
Distance traveled to reach target (cm)
WT (n = 16)
Camk2a HET KO (n = 20)
Probe trial 2 (30 days after the last training session)
D
p = 0.2506
Number of errors
E
p = 0.0834
Latency to reach target (s)
F
p = 0.0964
Distance traveled to reach target (cm)
