# Supplementary material for: Remote spatial memory deficits in mouse models of neuropsychiatric disorders with immature dentate gyrus phenotype
Source: Int J Neuropsychopharmacol. 2025 Aug 23;28(10):pyaf062. doi: 10.1093/ijnp/pyaf062 (PMC12553137; doi:10.1093/ijnp/pyaf062)
Supplement: Figure_S2_pyaf062 [file figure_s2_pyaf062.pdf]

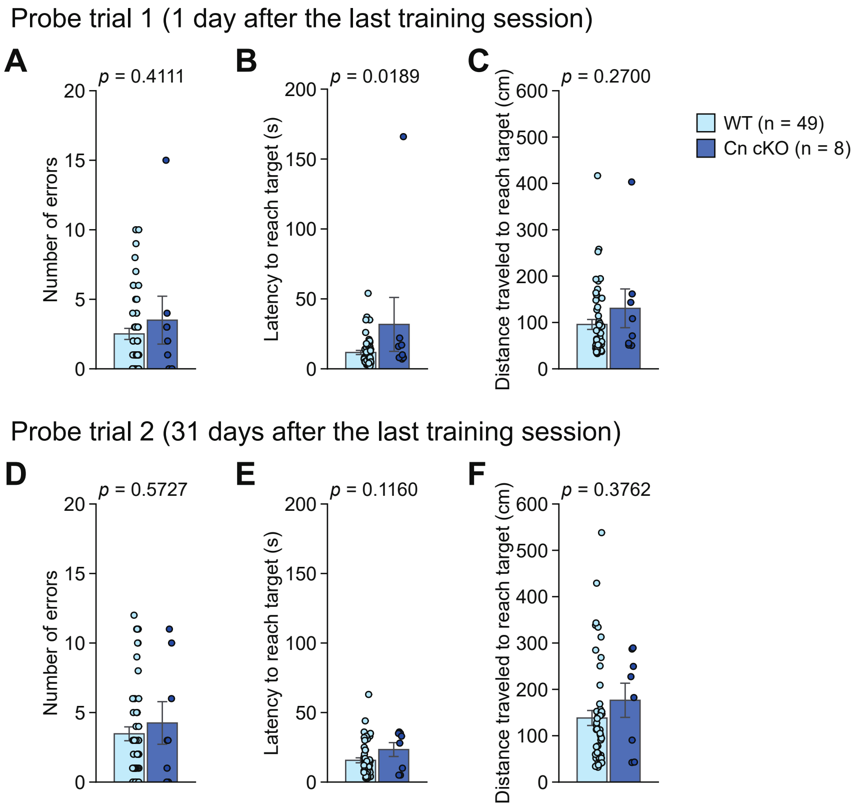
Probe trial 1 (1 day after the last training session)
A
p = 0.4111
Number of errors
B
p = 0.0189
Latency to reach target (s)
C
p = 0.2700
Distance traveled to reach target (cm)
WT (n = 49)
Cn cKO (n = 8)
Probe trial 2 (31 days after the last training session)
D
p = 0.5727
Number of errors
E
p = 0.1160
Latency to reach target (s)
F
p = 0.3762
Distance traveled to reach target (cm)
