# Supplementary material for: Remote spatial memory deficits in mouse models of neuropsychiatric disorders with immature dentate gyrus phenotype
Source: Int J Neuropsychopharmacol. 2025 Aug 23;28(10):pyaf062. doi: 10.1093/ijnp/pyaf062 (PMC12553137; doi:10.1093/ijnp/pyaf062)
Supplement: Figure_S4_pyaf062 [file figure_s4_pyaf062.pdf]

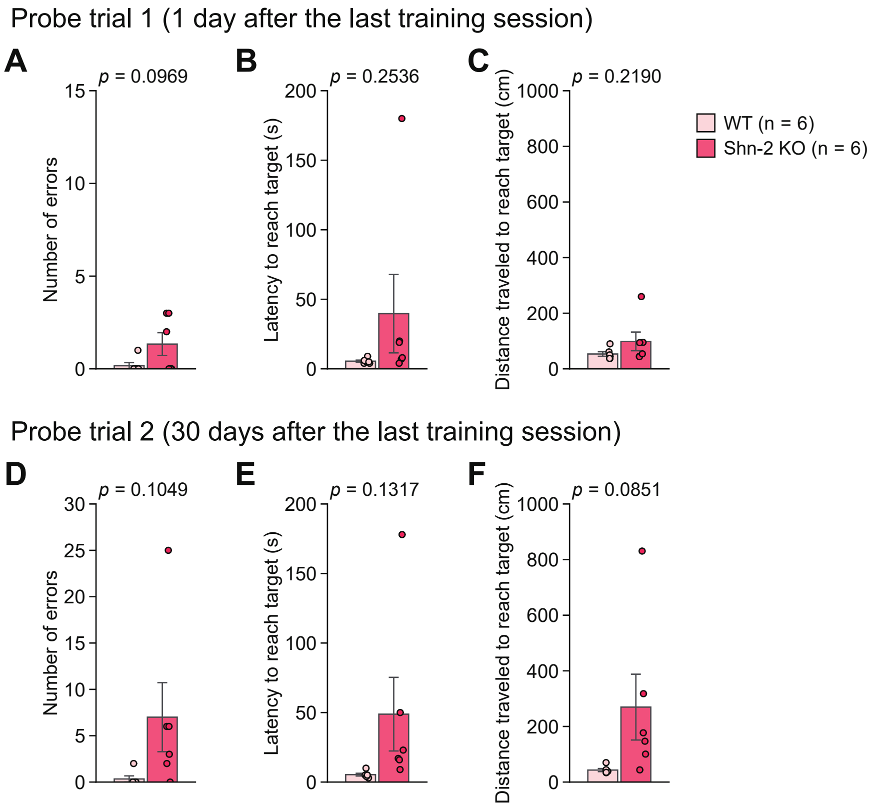

Probe trial 1 (1 day after the last training session)
A
p = 0.0969
Number of errors
15
10
5
0
B
p = 0.2536
Latency to reach target (s)
200
150
100
50
0
C
p = 0.2190
Distance traveled to reach target (cm)
1000
800
600
400
200
0
WT (n = 6)
Shn-2 KO (n = 6)
Probe trial 2 (30 days after the last training session)
D
p = 0.1049
Number of errors
30
25
20
15
10
5
0
E
p = 0.1317
Latency to reach target (s)
200
150
100
50
0
F
p = 0.0851
Distance traveled to reach target (cm)
1000
800
600
400
200
0
