# Supplementary figures and images for: Remote spatial memory deficits in mouse models of neuropsychiatric disorders with immature dentate gyrus phenotype
Source: Int J Neuropsychopharmacol. 2025 Aug 23;28(10):pyaf062. doi: 10.1093/ijnp/pyaf062 (PMC12553137; doi:10.1093/ijnp/pyaf062)

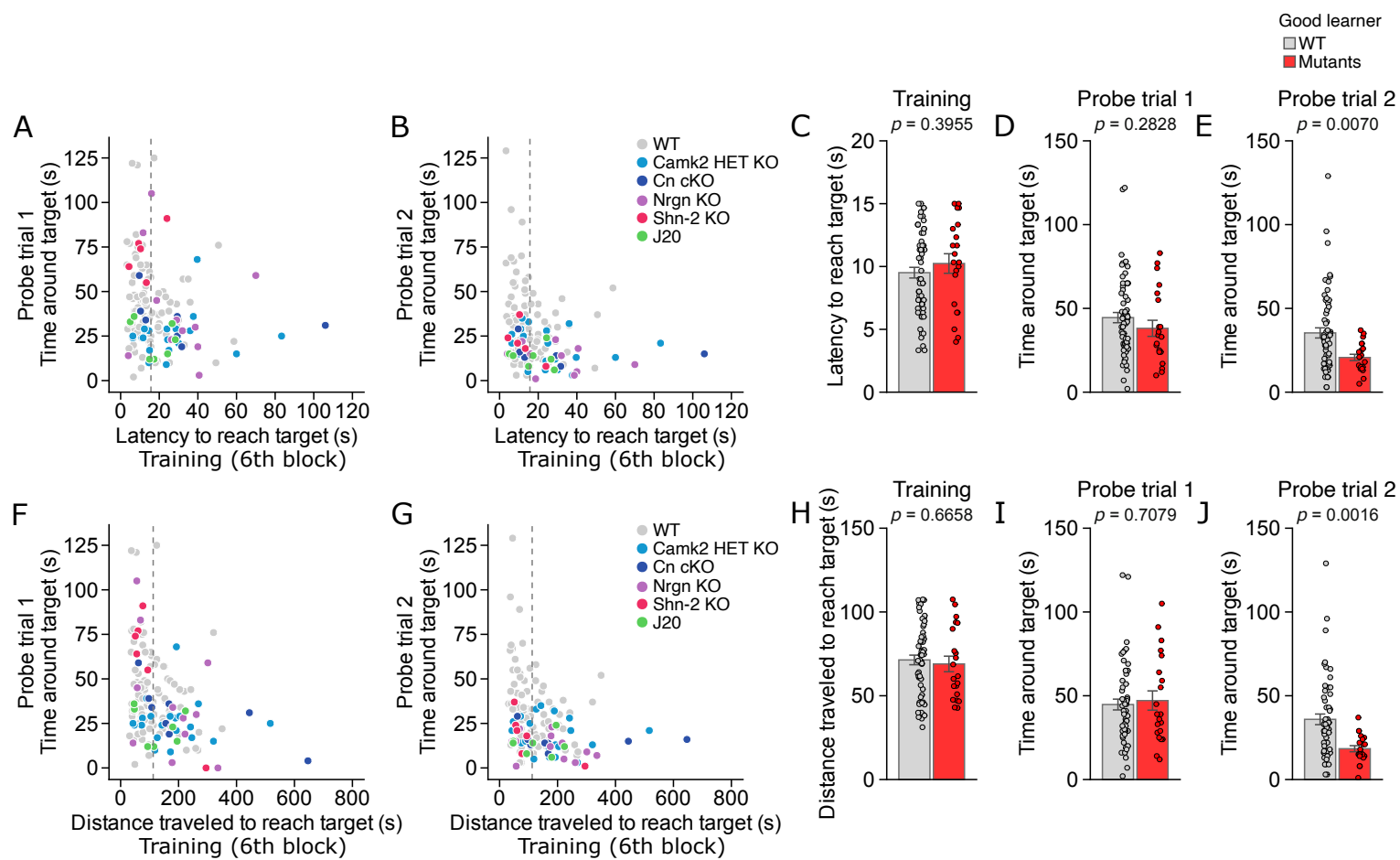

Supplement: Figure_S6_pyaf062 [file figure_s6_pyaf062.pdf]
