# Supplementary material for: Remote spatial memory deficits in mouse models of neuropsychiatric disorders with immature dentate gyrus phenotype
Source: Int J Neuropsychopharmacol. 2025 Aug 23;28(10):pyaf062. doi: 10.1093/ijnp/pyaf062 (PMC12553137; doi:10.1093/ijnp/pyaf062)
Supplement: Supplementary_Text_pyaf062 [file supplementary_text_pyaf062.docx]

**Supplementary Figure 1. Behavior in the probe trials of the Barnes maze test in Camk2a heterozygous knockout mice**

(A–F) Probe trials in Camk2a HET KO mice (n = 20) and WT mice (n =16). In the first and second probe trials conducted one day and 30 days after training session, (A, D) number of errors, (B, E) latency to reach the target hole (s), and (C, F) distance traveled to first reach the target hole (cm).

**Supplementary Figure 2. Behavior in the probe trials of the Barnes maze test in forebrain-specific Calcineurin knockout mice**

(A–F) Probe trials in Calcineurin (Cn) cKO mice (n = 8) and WT mice (n = 49). In the first and second probe trials conducted one day and 31 days after training session, (A, D) number of errors, (B, E) latency to reach the target hole (s), and (C, F) distance traveled to first reach the target hole (cm).

**Supplementary Figure 3. Behavior in the probe trials of the Barnes maze test in Neurogranin knockout mice**

(A–F) Probe trials in Neurogranin (Nrgn) KO mice (n = 12) and WT mice (n = 12). In the first and second probe trials conducted one day and 28 days after training session, (A, D) number of errors, (B, E) latency to reach the target hole (s), and (C, F) distance traveled to first reach the target hole (cm).

**Supplementary Figure 4. Behavior in the probe trials of the Barnes maze test in Schnurri-2 knockout mice**

(A–F) Probe trials in Schnurri-2 (Shn-2) KO mice (n = 6) and WT mice (n = 6). In the first and second probe trials conducted one day and 30 days after training session, (A, D) number of errors, (B, E) latency to reach the target hole (s), and (C, F) distance traveled to first reach the target hole (cm).

**Supplementary Figure 5. Behavior in the probe trials of the Barnes maze test in J20 mice**

(A–F) Probe trials in J20 mice (n = 7) and WT mice (n = 10). In the first and second probe trials conducted one day and 30 days after training session, (A, D) number of errors, (B, E) latency to reach the target hole (s), and (C, F) distance traveled to first reach the target hole (cm).

**Supplementary Figure 6. Remote memory deficit in good learners of mutant mice with immature dentate gyrus phenotype**

The scatter plots of the latency to reach the target hole (A, B) and distance traveled (F, G) in the last training session and time spent around the target hole at the probe trials 1 (A and F: for latency and distance in wild-types, r = -0.12, p = 0.2374 and r = -0.22 , p = 0.0366, respectively; for latency and distance in mutants, r = -0.36, p = 0.0083 and r = -0.39, p = 0.0038, respectively) and probe trial 2 (B: for latency and distance in wild-types, r = -0.19, p = 0.0680 and r = -0.27, p = 0.0093, respectively; for latency and distance in mutants, r = -0.28, p = 0.0396 and r = -0.21, p = 0.1391, respectively). The dotted lines indicate the mean value of the latency (A, B; mean = 15.71) and distance (F, G; mean = 112.39) in wild-type control mice of the five mutant strains. Mice showing the latency or distance traveled to reach the target hole shorter than the mean values were assigned as a good learner. (C, H) The latency and distance to reach the target hole (latency, t_80_ = 0.85, p = 0.3955; distance, t_76_ = 0.43, p = 0.6658), (D, I) time spent around the target hole at the probe trial 1 (latency, t_80_ = 1.08, p = 0.2828; distance, t_76_ = 0.38, p = 0.7079), and (E, J) time spent around the target hole at the probe trial 2 (latency, t_80_ = 2.77, p = 0.0070; distance, t_76_ = 3.27, p = 0.0016) in the good learners of mutants and wild-type controls.

**Supplementary Table 1. Statistical results of Barnes maze test**

**Supplementary Text**

**Materials and methods**

**Animals**

Five mutant strains of male mice, Camk2a heterozygous (HET) knockout (KO), forebrain-specific Calcineurin (CNB1, Cn) homozygous KO, Neurogranin (Nrgn) homozygous KO, Schnurri-2 (Shn-2) homozygous KO mice, J20 transgenic mice, and their wild-type (WT) control mice were used.

Camk2a HET KO mice were obtained from The Jackson Laboratory (strain B6.129P2-Camk2atm1Sva/J, JAX stock no 002362; Bar Harbor, ME, USA). Male HET KO mice were maintained by crossing with female C57BL/6J mice (Jackson Laboratory Japan, Inc., Kanagawa, Japan) for more than 6 generations. Wild-type mice obtained by crossing HET KO males and WT females were used as control mice (Camk2a HET KO, n = 20; WT, n = 16; 2.7–4.9 months of age at the start of behavioral testing).

Cn cKO mice were obtained by crossing a floxed CNB1 mice, provided by Dr. Gerald R Crabtree (Stanford University, CA, USA), and the forebrain-specific, α-CaMKII promoter-driven Cre mice (derived from mouse line of Tsien et al.). The floxed CNB1 and Cre mice were backcrossed with C57BL/6J for six generations after arrival at our laboratory. We used the mutant mice (flox/flox;Cre/wt: Cn cKO, n = 8) and three types of control mice (wt/wt;wt/wt, n = 15; wt/wt;Cre/wt, n = 19; flox/flox;wt/wt, n =15: WT, n = 49 in total) with a C57BL/6J background (6.2–9.8 months of age at the start of behavioral testing). There were no statistically significant differences in any behavioral measures during the training session of the Barnes maze test among the three types of control mice; therefore, the data were pooled.

Nrgn KO mice were gifted from Dr. Kuo-Ping Huang (Eunice Kennedy Shriver National Institute of Child Health and Human Development, MD, USA), which had been backcrossed to C57BL/6J for more than 10 generations. For behavioral experiments, KO and WT mice were obtained by crossing male heterozygous and female heterozygous mice (Nrgn KO, n = 12; WT, n = 12; 2.7–5.7 months of age at the start of behavioral testing).

Shn-2 KO mice were backcrossed with C57BL/6J mice (Jackson Laboratory Japan, Inc., Kanagawa, Japan) or BALB/cAJcl mice (CLEA Japan, Inc., Tokyo, Japan) for more than 10 generations. F1 hybrids obtained by mating heterozygous mice with C57BL6/J background and heterozygous mice with BALB/cAJcl background were used for this study (Shn-2 KO, n = 6; WT, n = 6; 2.2–5.2 months of age at the start of behavioral testing).

J20 mice with a C57BL/6J background (#034836-JAX; B6.Cg-Zbtb20Tg(PDGFB-APPSwInd)20Lms/2Mmjax), originally generated by Dr. Lennart Mucke (Gladstone Institute of Neurological Disease, UC San Francisco, CA, USA), were used. J20 and WT mice were produced using in vitro fertilization (IVF) and embryo transfer techniques. Fertilized eggs were obtained by crossing J20 males with C57BL/6J females (Jackson Laboratory Japan, Inc., Kanagawa, Japan) and transferred to the pseudo-pregnant ICR females (CLEA Japan, Inc., Tokyo, Japan). The pups were born to and reared by the ICR mothers (J20, n = 7; WT, n = 10; 5.3–5.6 months of age at the start of behavioral testing).

Camk2a HET KO mice and their wild-type mice were singly housed in plastic cages (25.0 × 18.2 × 13.9 cm) with paper bedding (PaperClean; Japan SLC, Inc., Shizuoka, Japan) after weaning because the mutants would show escalated aggression, possibly resulting in death of wild-type mice when the two genotypes of mice would be group-housed. The other strains of mice were housed in groups (two to four per cage) in plastic cages (22.7 × 32.3 × 12.7 cm, for Cn and Shn-2 strains; 25.0 × 18.2 × 13.9 cm, for Nrgn and J20 strains) with paper bedding. The room was illuminated with a 12-hour light/dark cycle (lights on at 7:00). The room temperature was maintained at 23 ± 2°C. The mice were given food (CRF‐1, Oriental Yeast Co., Tokyo, Japan) and water ad libitum throughout the study. All experiments were conducted under approval of the Institutional Animal Care and Use Committee of Fujita Health University.

**Barnes maze task**

Mice were moved to the sound-attenuated testing room (207.5 × 207.5 × 216.0 cm, interior dimensions; O’Hara & Co., Tokyo, Japan) and were placed in a rack covered with paper towels to avoid direct lighting from fluorescent ceiling lights in the room. The animals were habituated for at least 30 min before the start of the test. The Barnes maze test was performed between 9:00 and 18:00. The Barnes maze, originally developed by Carol Banes, is a dry, white circular surface (100 cm diameter) with 12 holes equally spaced around the perimeter (O’Hara & Co., Tokyo, Japan). The circular open field was elevated 75 cm above the floor. The apparatus was placed in the sound-attenuated room and illuminated by fluorescent light mounted on its ceiling. The illumination level was approximately 850 lx at the center of the field. A variety of fixed extra-maze clues surrounded the apparatus. A black Plexiglas escape box (17 × 13 × 7 cm) was placed under one of the holes (target hole) analogous to the hidden platform in the Morris water maze task. The location of the target was consistent for a given mouse, but randomized across mice. In the training session, the mice were placed in the center of the field and allowed to explore the maze freely. When the mice entered the escape box via the target hole or when 5 min had elapsed, they were left undisturbed in the box for 30 s. The training session was conducted with one to three trials per day (18 trials in total). The maze was rotated daily, with the spatial location of the target unchanged with respect to visual room cues, to prevent bias based on olfactory or proximal cues within the maze. Behaviors were recorded by a video camera mounted on the ceiling, and in each trial, the number of errors before first reaching the target hole, latency to reach the target hole (s), distance traveled to reach the target hole (cm), and the number of omission (defined by the visit to the target hole without subsequent entry into the target hole) were automatically calculated from the video images using a ImageJ-based software, ImageBM (see ‘Data analysis’). One day after the last training trial, a probe trial was conducted without the escape box for 180 s to assess recent memory (the first probe trial). Immediately after the first probe trial, mice were subjected to an additional trial with the escape box to avoid any possible interference with memory due to the probe trial. Following approximately 4 weeks (for Nrgn KO, 28 days; for Camk2a HET KO, Shn-2 KO, and J20, 30 days; for Cn cKO, 31 days), the probe trial was repeated for evaluating remote memory (the second probe trial). The mice were not subjected to any other tests between the first and second probe trials. In the probe trials, the time spent around each hole, number of errors before first reaching the target hole, latency to reach the target hole (s), and distance traveled to reach the target hole (cm) were calculated. To further assess spatial discrimination performance, the time spent around the target hole was compared with the time spent around adjacent holes (average time spent around the two holes positioned at both sides of the target hole) in each genotype. After each test, the apparatus was cleaned with hypochlorous acid water to eliminate olfactory cues.

**BrdU immunohistochemistry**

To assess neurogenesis in the subgranular zone of the DG in Nrgn KO mice, Shn-2 KO mice, and their WT control mice at two different ages during mature adulthood (for Nrgn, 3.9 and 7.1 months old; for Shn-2, 3.4–3.9 and 7.1–7.8 month old), mice received intraperitoneal injections of 5-bromo-2-deoxyuridine (BrdU; Sigma-Aldrich, St. Louis, MO, USA) dissolved in PBS every 2 h (four times per day) at a dose of 50 mg/kg body weight. Approximately 24 h after the last injection, the mice were deeply anesthetized and transcardially perfused with PBS followed by 4% PFA in 0.1M PB. Brains were removed and fixed in the same fixative at 4°C overnight. After post-fixation, the brains were soaked in 30% sucrose in PBS at 4°C for at least three days, embedded in Tissue-Tek OCT compound (Sakura Finetek Japan Co.,Ltd., Tokyo, Japan), and stored in a freezer at -80ºC. Brains were cut in 10-μm-thick coronal sections using a cryostat (CM1850/CM1950; Leica Biosystems, Wetzlar, Germany). The sections were mounted on slides, boiled in 10 mM citric acid for 30 min, washed three times with PBS, and blocked with 5% skim milk in PBS containing 0.1% Triton X-100 for 1 h at room temperature. The sections were incubated with a rat monoclonal antibody against BrdU (1:400; ab6326; Abcam, Cambridge, UK) at 4°C overnight, washed with PBS, and incubated for 1 h with Alexa Fluor 594-conjugated anti-rat IgG (1:1000; Invitrogen, MA, USA). Nuclear staining was performed with Hoechst33258 (Polyscience, Warrington, PA, USA). Images of the stained sections were obtained using a confocal microscope (LSM710; Zeiss, Göttingen, Germany). For quantitative analysis, at least five sections per animal were used to count the number of BrdU-positive cells in the granule cell layer of the DG. The cells were counted in a blind manner.

**Data analysis**

During the behavioral test, video images were captured at one frame per second. The images were analyzed using the ImageBM software developed by Tsuyoshi Miyakawa, which is based on the public domain ImageJ software (developed by Wayne Rasband at the National Institutes of Health and available at http://rsb.info.nih.gov/ij/). Using this software, the images were segmented into a black particle (mouse) and a white background (the maze surface). The position of the center of gravity of the black particles in the maze in each image was used to measure the time spent around each hole. The distance traveled was calculated from the distance between each set of x-y coordinates for the center of gravity of the black particle in consecutive images. Behavioral data were analyzed using Student’s *t*-test, paired *t*-test, and two-way repeated-measures ANOVA. The data from the BrdU assay were analyzed by Student’s *t*-test. The significance level was set at p < 0.05. Statistical analyses were performed using SAS University Edition software (SAS Institute Inc., NC, USA).
